# Supplementary material for: A synthetic ERFVII-dependent circuit in yeast sheds light on the regulation of early hypoxic responses of plants
Source: Proc Natl Acad Sci U S A. 2026 Mar 10;123(11):e2524358123. doi: 10.1073/pnas.2524358123 (PMC12994171; doi:10.1073/pnas.2524358123)
Supplement: Supplementary file 1 — Appendix 01 (PDF) [file pnas.2524358123.sapp.pdf]

Supporting Information for

A synthetic ERFVII-dependent circuit in yeast sheds light on the regulation of early hypoxic responses of plants

Mikel Lavilla-Puerta, Yuming He, Luca Piccinini, Lorenzo Di Paco, Antonis Papachristodoulou, Francesco Licausi, Beatrice Giuntoli

Corresponding authors: Francesco Licausi and Beatrice Giuntoli

Emails: francesco.licausi@biology.ox.ac.uk, beatrice.giuntoli@unipi.it

This PDF file includes:

Supporting text

Figures S1 to S12

Tables S1 to S12

SI References

Supporting Information Text

Extended methods

Plant model description

Gene expression analysis from Col-0 seedlings growing on vertical plates subjected to 2 h hypoxia in **Fig. 1** was fitted to a logistic equation with the following formula:

$$y(t) = \frac{A}{1 + e^{-k(t-t_{half})}}$$

where $y(t)$ is the relative mRNA expression at time t, A is the upper asymptote (maximum level of $y(t)$), k is a constant related to the steepness of the curve, and t_{half} is the time at which $y(t)$ is $A/2$ and is also the point where the curve grows most rapidly. All values for A, k and t_{half} were fitted to each of the nine HRGs displayed in **Fig. 1** using MATLAB's `lsqcurvefit`, and are shown in **SI Appendix, Table S1**.

To quantify the speed of HRG response, we defined response time (RT) as $t_{90\%}$ - $t_{10\%}$, representing the time interval required for expression to rise from 10% ($t_{10\%}$) to 90% ($t_{90\%}$) of the maximum. We then applied a linear mixed-effects model using MATLAB's `fitlme` function, to test whether the density of HRPE elements within the promoters were associated with the RT, while also accounting for scaled promoter length (hereafter denoted as $PromoterLength$):

$$RT_{ij} = \beta_0 + \beta_1 \cdot HRPEDensity_{ij} + \beta_2 \cdot PromoterLength_{ij} + u_j + \varepsilon_{ij}$$

Here β_0 is the intercept, β_1 and β_2 are fixed effects of HRPE density (number of HRPE motifs divided by the total promoter length in base pairs) and scaled promoter length, u_j is the random effect capturing gene-specific variation, and ε_{ij} is the residual error. Because promoter lengths vary substantially among HRGs, we standardized them using z-score normalization:

$$PromoterLength_{ij} = \frac{P_{ij} - \mu}{\sigma}$$

where P_{ij} is the promoter length, μ is the mean of the promoter lengths across all HRGs, and σ is the corresponding standard deviation of the promoter lengths. This scaling places both predictor variables (HRPE density and promoter length) on comparable scales, improving interpretability and numerical stability of the regression estimates. In R, we used the `DominanceAnalysis` implemented in (1, 2) to assess the relative contribution of HRPE density and promoter length to the response time, following. The code developed for the plant model description and the dominance analysis in R were uploaded on GitHub: *GitHub - ymiplant/synthetic-erfvii-yeast-hypoxia*.

Yeast transformation

Yeast was transformed according to (3), with minor modifications. Prior to transformation, wild-type cells were grown at 30°C, 150 rpm on YPDA containing 20 g L^{-1} peptone, 10 g L^{-1} yeast extract, 20 g L^{-1} of glucose (Duchefa) and 20 mg L^{-1} adenine hemisulfate (Sigma-Aldrich), supplemented with 20 g L^{-1}

agar (Duchefa). Single colonies were grown to saturation (overnight) in 5 ml of liquid YPD, at 30°C under continuous shaking at 150 rpm, then diluted 1:100 in 50 mL liquid YPD and grown for 6 additional hours. Cells were then harvested by centrifugation at 5000 g, 5 min and washed three times with sterile water. For each transformation, 100 µL of cell suspension were centrifuged and resuspended in a buffer containing 36% PEG 3350, 100 mM lithium acetate and 100 µg single-strand DNA, plus 1 µg of plasmid DNA. Integrative plasmids were first linearized by restriction for 2 h at 37°C, with Anza endonucleases (Thermo-Fisher Scientific): pAG305-, pAG303- and pAG304-based plasmids were linearized with BstXI (cat. IVGN0666), pAG306-based plasmids with ApaI (cat. IVGN0326). Cells were incubated at 42°C for 60 min. Transformed cells were collected by centrifugation and plated on the appropriate solid SD medium (20 g L^{-1} agar) for selection. Positive transformants were transferred to liquid SD, prior to further transformations or experiments. All yeast strains used in this work are listed in **SI Appendix, Table S6**.

Cloning of constructs for yeast and plant expression

Some of the Gateway-compatible destination vectors listed were purposely generated for this study, by modification of the existing destination plasmids for yeast expression. In detail, pAG304NLUC was generated from pAG304GPD-ccdb (Addgene, #14135) by restriction and ligation of a synthetic ccdb-NLUC string (GeneArt, Thermo-Fisher Scientific, sequence in **SI Appendix, Table S7**) flanked by SacI and XhoI restriction sites. pAG304GPD-ccdb and ccdb-NLUC were cut with SacI and XhoI (cat. IVGN0208 and IVGN0086, Anza, Thermo-Fisher Scientific) and joined using the Anza T4 DNA ligase (cat. IVGN2104, Thermo-Fisher Scientific). The destination plasmids pAG303HRPE$_{ADH}$ and pAG305HRPE$_{ADH}$ were obtained from pAG303GPD and pAG305GPD backbones (Addgene, #14135 and #14138, respectively), produced upon removal of P*GPD* promoter sequence following a digestion with SacI and XbaI (IVGN0126, Anza, Thermo-Fisher Scientific). The promoter was replaced by ligation with an HRPE$_{ADH}$ sequence, amplified from HRPE$_{ADH}$_pE (**SI Appendix, Table S9**) with compatible SacI and XbaI extremities incorporated in the PCR primers (**SI Appendix, Table S8**). The destination vector pBGWNLUC7 was, instead, obtained from pBGWL7 (4) and used to generate the binary construct HRPE$_{ADH}$-NLUC-pBGWNLUC7 for stable plant transformation. A DNA string containing the SacI-attR1-ccdB-attR2-NanoLuc-HindIII sequence was synthesized (GeneArt, Thermo-Fisher Scientific; **SI Appendix, Table S7**) and recombined into the pBGWL7 upon restriction with SacI and HindIII (IVGN0168, Invitrogen) and ligation with Anza T4 DNA ligase. Recombinant plasmids were propagated *E. coli* One Shot *ccdB* Survival 2 T1^R Competent Cells (Thermo-Fisher Scientific), before use in LR reactions.

PCO4-SV40 was cloned from 7-day-old Arabidopsis seedling cDNA with two subsequent PCR reactions, using two overlapping primers (**SI Appendix, Table S8**) to incorporate a C-terminal SV40 domain (PKKKRKV) into the PCO4 coding sequence. To generate the RAP2.12-derived chimeric transcription factors, the full RAP2.12 coding sequence was amplified from the same cDNA template as above and fused to different 3' fragments by overlapping PCR. Specifically, RAP2.12 coding sequence was amplified from Arabidopsis cDNA from seedlings and fused to a 6xTAL-4xVP16 fragment amplified from the plasmid dCas9-TV (5) to obtain RAP2.12-6TVP. The fusion sequence was created by overlapping PCR of fragments amplified with a combination of RAP2.12_Fw and RAP2.12-6TVP overlapping primers from **SI Appendix, Table S8**. Similarly, a Gal4 AD was amplified from the plasmid pDEST22 (Thermo-Fisher Scientific) and fused to RAP2.12 to obtain the RAP2.12-GAL4AD insert. In the

case of SYRAP, finally, a chimeric GAL4Ste sequence (*$GAL4_{1\text{-}147}$-$Ste12_{301\text{-}335}$-$GAL4_{148\text{-}196}$*) was fused, using SYRAP overlapping primers. The GAL4Ste template (6) was provided by a synthetic NLUC-UBQ-$RAP2.12_{2\text{-}50}$-GAL4STE string (**SI Appendix, Table S7**), purchased from GeneArt (Thermo-Fisher Scientific). The ubiquitin fusion factor UbSYRAP was further amplified from the entry plasmid UbSYRAP_201 SYRAP. A UBQ fragment was amplified from the same string DNA template, with SY_UBQ_Fw and OL_SY_Rv primers, and combined with a SYRAP fragment amplified with OL_SY_Fw and G4Ste_Rv (**SI Appendix, Table S7 and S9**) UbSYHRE was generated by overlapping PCR between the synthetic templates UBQ-HRE2 and GAL4Ste (**SI Appendix, Table S8**).

All above mentioned constructs were made BP-compatible thanks to the presence of BP adaptors in their flanking sequences. The inserts were subsequently amplified with primers BP_Ad_Fw and BP_Ad_Rv (**SI Appendix, Table S8**), to restore full attB sites, and recombined into pDONR201 using the Gateway™ BP Clonase™ II Enzyme mix (Thermo-Fisher Scientific).

UAS_{GAL} activation experiments in MaV203 strains

Overnight cultures were diluted to OD_{600} = 0.1 and grown for 6 h. Growth rates were calculated from optical density values of the microcultures, measured using the Multiskan Go 1510 Sky plate reader (Thermo-Fisher Scientific). To overcome the low saturation limit of optical detectors in microplate readers, the OD_{600} values were corrected by means of an empirical formula (7), where (corrected OD_{600}) = $-8.7202(OD_{600})^2 + 11.363(OD_{600})$. Cells were then diluted to OD_{600} = 0.1 and re-incubated at 30°C, to follow their growth at 2 h intervals. Growth rate (k) was calculated between 4 and 6 h after dilution, when positive controls showed a clear exponential growth phase (**SI Appendix, Fig. S6A**), according to the following formula (8):

$$k = \frac{log_{10}\left(\frac{corrected\ OD_{600,6h}}{corrected\ OD_{600,4h}}\right)}{0.301 \cdot 2}$$

Β-galactosidase (LacZ) activity from the P*GAL1-LacZ* reporter was measured by adapting to yeast a colorimetric assay initially developed for animal tissues (9). Approximately $2x10^7$ cells were collected by centrifugation, washed twice with sterile water, once with PBS (137 mM NaCl, 2.6 mM KCl, 10 mM Na_2HPO_4, 1.8 mM KH_2PO_4; pH=7,4) and resuspended in a buffer composed of 0.25 M Tris-HCl, 2.5 M EDTA, 0.2 M lithium acetate, 1% v/v Triton X-100 and 4.2 µl mL^{-1} antifoam (Merck) (pH=7.4). Cell suspensions were flash frozen in N_2 and then homogenized with a Bead Mill 4 Homogenizer (Fisher Scientific), using two cycles of 120 sec at maximum speed. For the colorimetric assay, 100 µl of the lysate were mixed with 900 µl of CPRG solution containing 25 mM MOPS, 100 mM NaCl, 10 mM $MgCl_2$ (pH=7.5) and 84 mg l^{-1} of red-β-d-galactospyranoside (CPRG) substrate (Merck). Samples were incubated at 37°C and monitored until a color shift from orange to red became visible (8 or 24 h), indicative of substrate hydrolysis. CPRG absorbance at 575 nm was measured in a spectrophotometer and LacZ activity was calculated according to (9).

To prepare spots, 10^5 cells were collected, washed with 1 ml sterile water four times and diluted on four 1:10 serial dilutions. Suspensions were spotted in 10 µl volume on -leu-trp SD solid media supplemented with 10 mM 3-amino-1,2,4-triazole (3-AT, Merck), found to be the optimal concentration to overcome the leaky HIS3 activity in the negative controls (EV/RAP2.12), while allowing growth of the positive controls expressing GAL4Ste. Plates were incubated for 3 days at 30°C either in normoxic or hypoxic conditions and scanned.

Immunoblotting

Total protein samples were extracted from 2-3x10^8 yeast cells in late exponential cultures (10). Arabidopsis seedling extracts were prepared according to (11). A positive control for GAL4 immunoblottings was provided by a recombinant protein, produced *in vitro* via the TnT Coupled Wheat Germ Extract Systems (Promega). To this end, the NLUC-UBQ-RAP2.12$_{2\text{-}50}$-GAL4Ste string (**SI Appendix, Table S7**) was LR cloned into the pF3A WG (BYDV) Flexi Vector (Promega) and *in vitro* transcription and translation was performed, following the manufacturer's recommendations.

Proteins were separated via SDS-PAGE on 10% polyacrylamide gels (NuPage Bis-Tris Gels, Thermo-Fisher Scientific) and transferred to poly-vinylidene difluoride membranes (Bio-Rad), as described before (12). Yeast blots were hybridized with primary monoclonal GAL4-DBD (sc-577) or α-tubulin antibodies (sc-53030, Santa Cruz Biotechnologies), used at 1:1000 and 1:5000 respectively. Plant blots were hybridized with the primary monoclonal antibodies anti-HA (#26183, Thermo Fisher Scientific, 1:1000) and anti-actin11 (AS13 2640, Agrisera 1:2000), or the polyclonal antibodies anti-ADH and anti-PDC (AS10 685 and AS10 691 respectively, Agrisera 1:1000). HRP-conjugated secondary antibodies were used for immunodetection. A polyclonal anti-mouse IgG (BioVision, cat. no. 6402-05, 1:20000) was used against GAL4-DBD and HA; anti-Rat IgG (AS10 1187, Agrisera, 1:10000) against α-tubulin, and anti-Rabbit IgG (AS09 602, Agrisera, 1:10000) for actin, ADH and PDC. Bands were visualized with a Clarity chemiluminescent substrate (1705060, Bio-Rad), using a ChemiDoc MP Imaging System (Bio-Rad). Protein loading on the gels was evaluated by membrane staining with 0.1% amido black 10-B (12). Band intensity quantification was performed with the ImageLab® software (Bio-Rad).

Yeast model description and assumptions

To build the four Ordinary Differential Equation (ODE)-based models illustrated in **Fig. 6A**, we incorporated both Michaelis-Menten Kinetics and Mass Action laws to describe the gene expression and biochemical reaction network processes respectively (**SI Appendix, Tables S3** and **S4**).

The basic model (GG) represents a single copy of a constitutively expressed RAP2.12 and PCO. Here, RAP2.12 (UbSYRAP) protein is synthesized at a rate k_1 and degraded at a rate k_2. Additionally, it undergoes degradation by the cysteine N-degron pathway, following Michaelis-Menten kinetic laws, at rates represented by k_3, K_{m1}, and K_{m2}. The activation of HRPE promoter, which is targeted by RAP2.12 depending on the binding rates of UbSYRAP to the HRPE promoter for NLUC/FLUC ($HRPE_1$), produces NLUC/FLUC activity following Michaelis-Menten kinetics at a rate represented by k_4 and Km_3 and NLUC/FLUC undergoes degradation at a rate of k_5. In the models with additional feedback loops involving PCO1 (GP), UbSYRAP (UG), and both (UP), the HRPEs are targeted by UbSYRAP depending on the binding rates of UbSYRAP to the HRPE promoter for the PCO1 ($HRPE_2$) and UbSYRAP ($HRPE_3$) feedback loop, respectively. Subsequently they are activated following Michaelis-Menten kinetics at rates represented by k_6 and k_9, respectively and Km_3. PCO1 has unique Michaelis-Menten kinetic parameters in N-degron pathway, different from PCO4, which are k_8, K_{m4} and K_{m5}. k_4, k_6 and k_9 are aggregated parameters overseeing all processes involved in protein synthesis rate.

To simplify our model, each reaction is assumed to take place in a spatially homogeneous environment, without considering compartmentalization or specific microenvironments within the cell and that each reaction species, including UbSYRAP, PCO1/4, oxygen, and NLUC, is evenly distributed. To concentrate on the oxidation process and simplify the model, we only modelled

UbSYRAP degradation after its oxidation by PCO1/4, assuming arginylation, ubiquitination, and degradation by ATE, PRT6, and the 26S proteasome happen immediately afterwards. Additionally, we assumed that the concentration of PCO4 has already reached a steady state during normoxia and remains constant even after applying the 1% hypoxia treatment. Thus, PCO4 can be considered as a parameter without introducing its own expression and degradation rates which simplifies and adds more flexibility to our model.

Non-linear fitting and simulation

We used MATLAB's 'Talwar' and 'Welsch' weight functions within the `nlinfit` function for robust fitting to re-fit the catalytic rate constants (k_8/k_3) for PCO1/4-mediated oxidation of RAP2.12 (UbSYRAP), the Michaelis-Menten constants (K_{m4}/K_{m1}) for O_2 as a substrate of PCO1/4, and the Michaelis-Menten constants (K_{m5}/K_{m1}) for UbSYRAP as a substrate of PCO1/4, respectively. This three-dimensional fitting, which is depicted in **SI Appendix, Fig. S11A**, was performed based on published RAP2.12 and O_2 kinetic data (13). The initial guesses for UbSYRAP protein synthesis rate (k_1) and degradation rate (k_2), and PCO4 concentration in yeast were obtained by simulation visualization using MATLAB's ode45 solver, and subsequently the parameters were fitted using the `lsqcurvefit` function, initialised by those guesses, based on the published yeast data with proper rescaling (12). (**SI Appendix, Fig. S11B**). Additionally, we applied the same rate for pGDP-regulated UbSYRAP production and degradation in all four different models. The remaining parameters in the models were fitted using the same method, based on the data shown in **Fig. 5C**. All parameters are listed in **SI Appendix, Table S5**.

To simulate hypoxic responses, the initial concentrations of UbSYRAP, PCO1, and mRNA as inputs were obtained from steady-state simulations conducted under normoxic conditions (21% O_2) for six hours (**SI Appendix, Tables S2 and S3**). Subsequently, dynamic simulations of hypoxic responses (NLUC/FLUC) and adjusted UbSYRAP (log10(UbSYRAP)/2) concentration were conducted under 1% O_2 conditions for four hours. The code used to generate the yeast-based models in MATLAB is available on Github (*GitHub - ymiplant/synthetic-erfvii-yeast-hypoxia*).

Data representation and statistics

Box plots represent median (line) and interquartile range (IQR), whiskers span from minimum to maximum values. Graphpad Prism v. 8.0.1 was adopted to depict data and to evaluate statistical significance, which passed a Shapiro-Wilk normality test ($p<0.05$) prior to the analysis. Pairwise comparisons through Student's t-test (n=4-6) were applied to data in Figure 1C, Figure 2B, Figure 3B and C, Figure 4 and Figure 5C, as well as in SI Appendix, Figures S3, S5A, S6B and C, S7, S8B and D, and S10. For the analysis on figures S8D and S10, separate analysis tables were generated as SI Appendix, Table S11 and Table S12. One-way Anova was used in Figures 2D, 3A and E, 5E and F and SI Appendix, S8E (in these last three, treatment-independent analysis). In both kinds of procedure, the multiple comparisons were corrected with a two-stage linear step-up procedure of Benjamini, Krieger and Yekutieli, with Q=1%. Statistical significance was depicted as asterisks (****, $p<0.0001$; ***, $0.0001\leq p<0.001$; **, $0.001\leq p<0.01$; *, $0.01\leq p<0.05$) or letters (for $p<0.05$, unless stated otherwise).

Two-way Anova was, instead, adopted in SI Appendix, Figures S5B, S8A and F, where the analysis was followed by a Tukey post-hoc test, with letters depicting significantly different groups for $p<0.05$.

Grey shadings in figures indicate statistical difference, according to multiple Student's t-test, between a given time point in hypoxia and the normoxic control (t_0). The first group to be significantly different from normoxia (t_0), regardless of further changes, was shaded in Figure 1A and 5B, and SI Appendix Figures S7A and C and S8B. Instead, where different shades of grey appeared, they depicted different significance levels corresponding to the accompanying asterisks (****, $p<0.0001$; ***, $0.0001\leq p<0.001$; **, $0.001\leq p<0.01$; *, $0.01\leq p<0.05$) in Figure 4C-F, 5C and SI Appendix, Figure S7B.

Supporting Information Figures

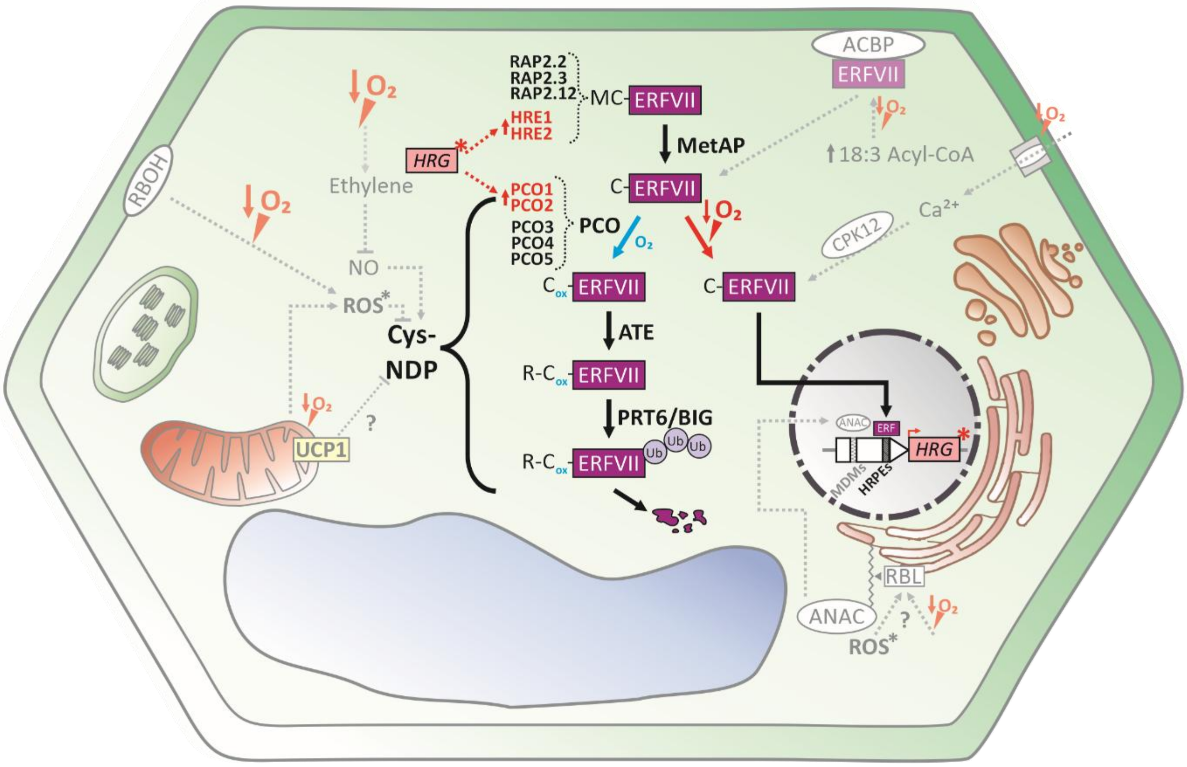

Figure S1. Simplified overview of plant hypoxic signaling events. The Cys-N degron pathway (Cys-NDP) exerts a main regulatory role over Ethylene Response Factors VII (ERFVIIs). Exposing an N-terminal cysteine (upon methionine removal by Methionine Aminopeptidases (MetAPs)). ERFVIIs have their N-terminal Cys further oxidised by Plant Cysteine Oxidases (PCOs), arginylated by Arginyl Transferases (ATEs) and later polyubiquitinated by ubiquitin ligases (PRT6 and BIG), prior to proteasomal degradation. Hypoxia (red triangle) inhibits PCO activity and allows ERFVIIs into the nucleus, binding Hypoxia Responsive Promoter Elements (HRPEs) and triggering hypoxia responsive gene (HRG) expression. Additionally, NAC domain-containing proteins (ANACs), cleaved by Rhomboid-like proteases (RBLs) from the ER in presence of ROS and/or hypoxia (process still unknown), induce HRG expression by binding to Mitochondrial Disfunction Motifs (MDMs). As main effectors, ERFVIIs are subject to additional regulation, both directly through their hypoxia-induced dissociation from Acyl-CoA Binding Proteins (ACBPs) at the plasma membrane, and phosphorylation via CPK12-mediated calcium signaling. Other mechanisms further attune the Cys-NDP: from the mitochondria, UCP1 (released as retrograde signal upon electron transport chain disruption), as well as reactive oxygen species (also generated at the plasma membrane) both inhibit the Cys-NDP. Nitric oxide (NO), a positive regulator of the pathway, is depleted by the ethylene accumulated during hypoxia. In this (simplified) network, ERFVII regulation through the Cys-NDP plays a central role in triggering hypoxic responses that positively feedback the expression of ERFVII members Hypoxia Responsive ERF (HRE) 1 and 2, as well as PCO1 and 2.

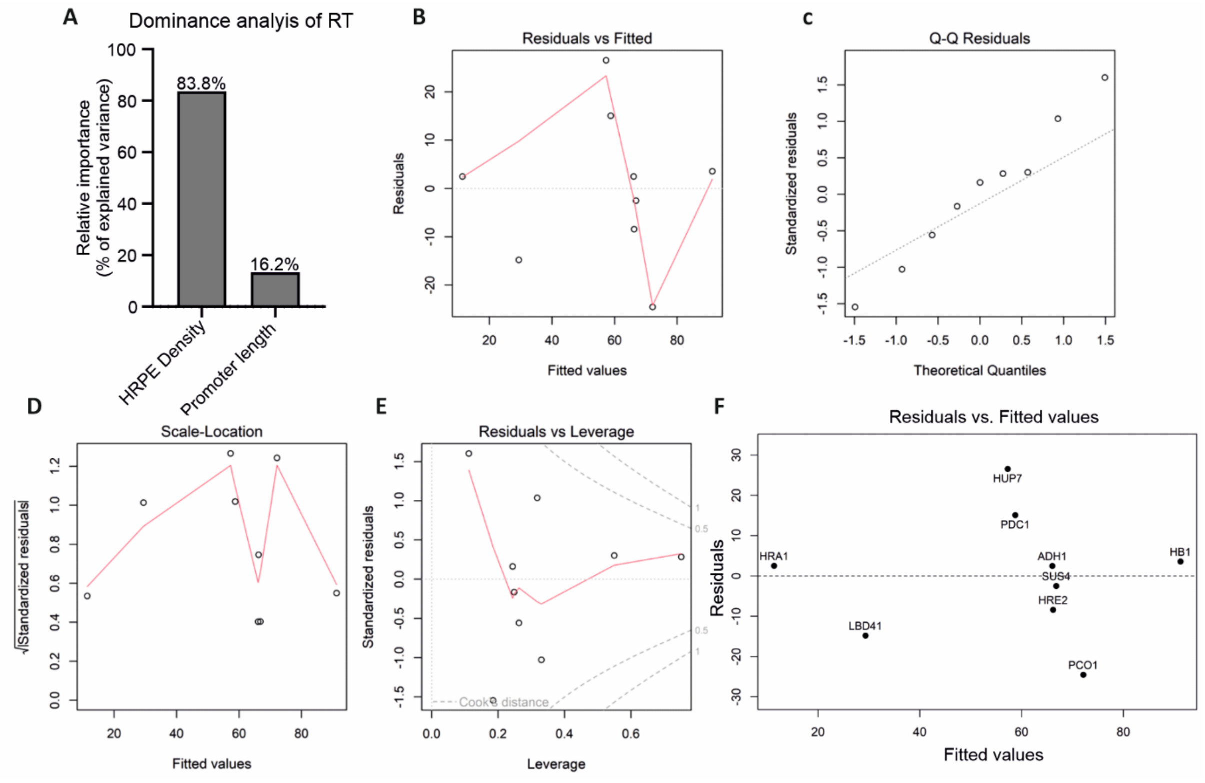

Figure S2. Dominance analysis of linear mixed-effects model in Fig. 1B. (A) Dominance analysis of the linear model. Bars show the relative importance of HRPE density and promoter length (z-scored), expressed as the percentage of the model R^2 attributable to each predictor (average contribution across all subset models). **(B-E)** Residual diagnostics for the linear model used in dominance analysis: **(B)** standard diagnostic plots for the linear model used in dominance analysis, **(C)** normal Q–Q plot of standardized residuals, **(D)** scale–location plot showing the square root of absolute standardized residuals versus fitted values, and **(E)** residuals versus leverage with Cook's distance contours (0.5 and 1). Red curves indicate LOESS smoothers to aid visual detection of systematic trends, dashed reference lines indicate zero residuals or theoretical expectations under the model, and Cook's distance contours highlight observations with potentially high influence. **(F)** Residuals vs fitted values for the fixed-effects linear model used in dominance analysis. Each point corresponds to one gene, and genes are shown next to points. The dashed horizontal line denotes zero residual.

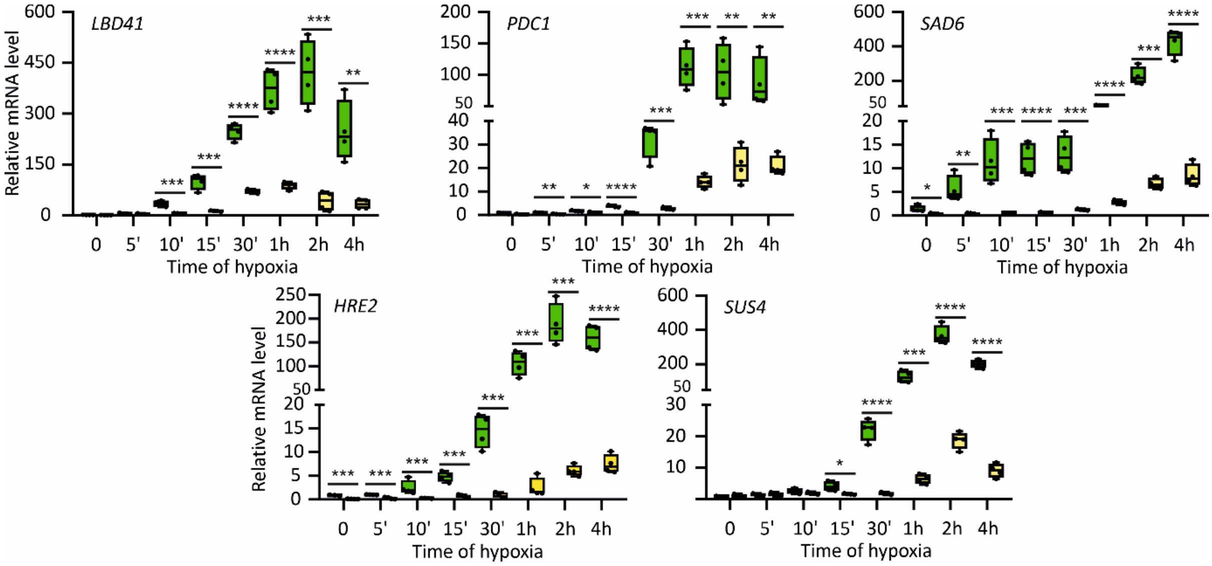

Figure S3. Profiling of hypoxia-responsive gene (HRG) expression in Arabidopsis *erfvii* mutants. *Lateral organ Binding Domain 41* (*LBD41*), *Pyruvate Decarboxylase 1* (*PDC1*), *Steraroyl-Acyl Carrier Protein 6* (*SAD6*), *Hypoxia Responsive ERF 2* (*HRE2*) and *Sucrose Synthase 4* (*SUS4*) transcript abundance (normalized to *UBQ10* and relative to an aerobic Col-0 sample) in *erfVII* and Col-0 7-day-old seedlings, exposed to dark hypoxia (1% O_2 v/v) at 23°C, for 4 hours. Asterisks indicate significant differences after pairwise comparisons between genotypes, for n=4 (****, p<0.0001; ***, 0.0001≤p<0.001; **, 0.001≤p<0.01; *, 0.01≤p<0.05).

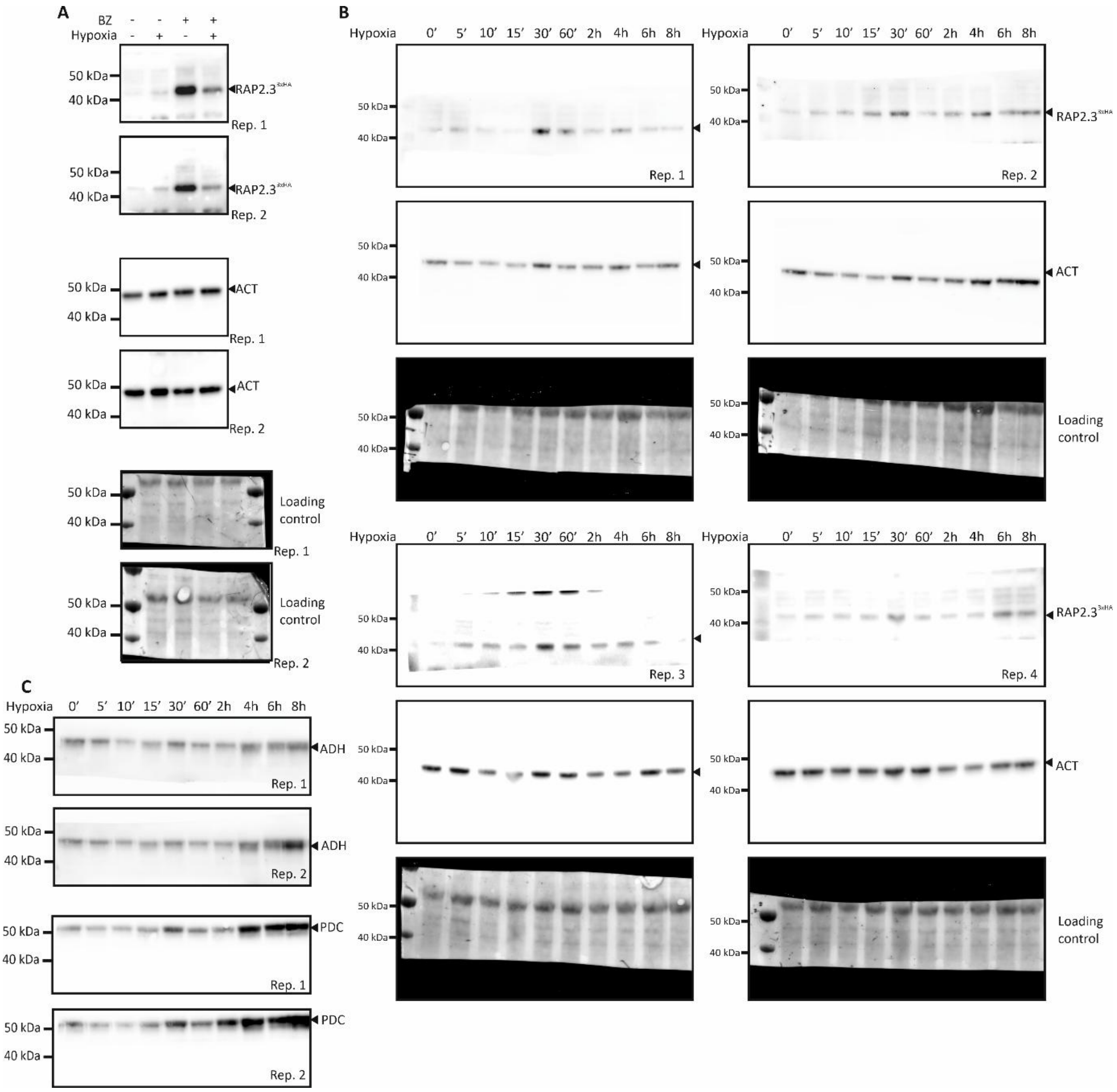

Figure S4. Uncropped blots from Arabidopsis protein samples. (A) Supporting blots for Figure 2B. Seedlings were grown in liquid medium for 7 days and then treated with 100 µM bortezomib (BZ) or 1% (v/v) DMSO. **(B)** Supporting blots for Figure 2D. Detection of HA-tagged RAP2.3, actin (ACT), and loading control (n=4) replicates. **(C)** Blots showing alcohol dehydrogenase (ADH) and pyruvate decarboxylase (PDC) in replicates 1 and 2 from panel (B). All proteins were detected from total protein extracts of 7-day-old Arabidopsis seedlings containing a *35S:RAP2.3*^{3xHA} transgene, grown in vertical plates and moved from normoxia (t_0) to hypoxia (1% O_2 v/v) for the indicated duration.

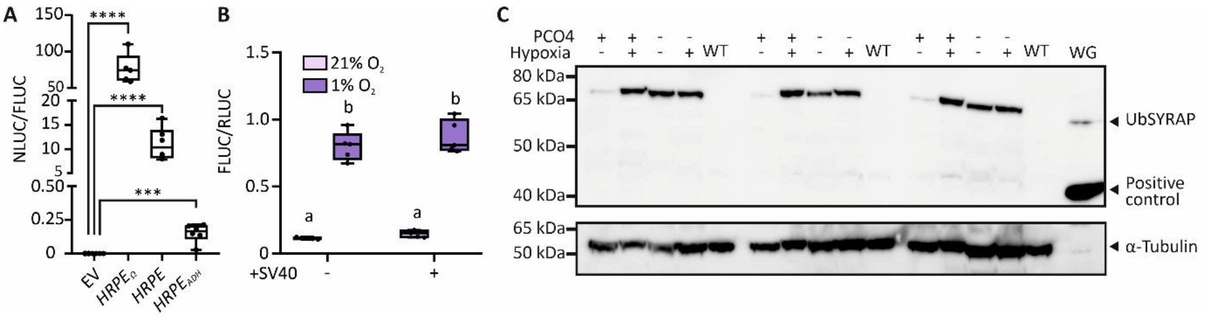

Figure S5. Characterization of the components of the yeast transcriptional circuit. **(A)** Basal NLUC activity of *HRPE-NLUC, $HRPE_{ADH}$-NLUC* and *$HRPE_{\Omega}$-NLUC* promoter modules, in comparison with an empty vector (EV) (n=5-6). Output signals are expressed as ratios between NLUC activity and FLUC activity from a reference *GPD (glyceraldehyde 3-phosphate dehydrogenase) promoter: FLUC* integrative construct. Output signals are expressed as activity ratios with FLUC encoded by a reference P*GPD:FLUC* integrative construct. **(B)** Activity of the SV40-tagged PCO4 version used in this study, expressed by the integrative plasmid pAG305 (14), on the model substrate DLOR (12). DLOR output is displayed as FLUC/RLUC ratio (n=5). For reference, the untagged PCO4 version used in (12), expressed by the episomal plasmid pAG415 (14) was included in the experiment. **(C)** Uncropped blot of yeast cells expressing UbSYRAP and PCO4 or GUS (-PCO4) treated with hypoxia. A triplicate experiment is shown. UbSYRAP was revealed with an α-GAL4BD antibody. α-Tubulin was used as housekeeping protein on the same stripped and hybridized membrane. WT, untransformed W303 cells. WG, wheat germ extract expressing a recombinant NLUC-UBQ-RAP2.$12_{2\text{-}50}$-GAL4STE12 protein, as positive control. (**SI Appendix, Table S7**). Asterisks in (A) represent statistically different groups, as tested via multiple Student's t-tests (n=6; ****, $p<0.0001$; ***, $0.0001 \leq p < 0.001$). Letters in (B) represent statistically different groups, as tested by two-way Anova, for n=5 and $p<0.05$.

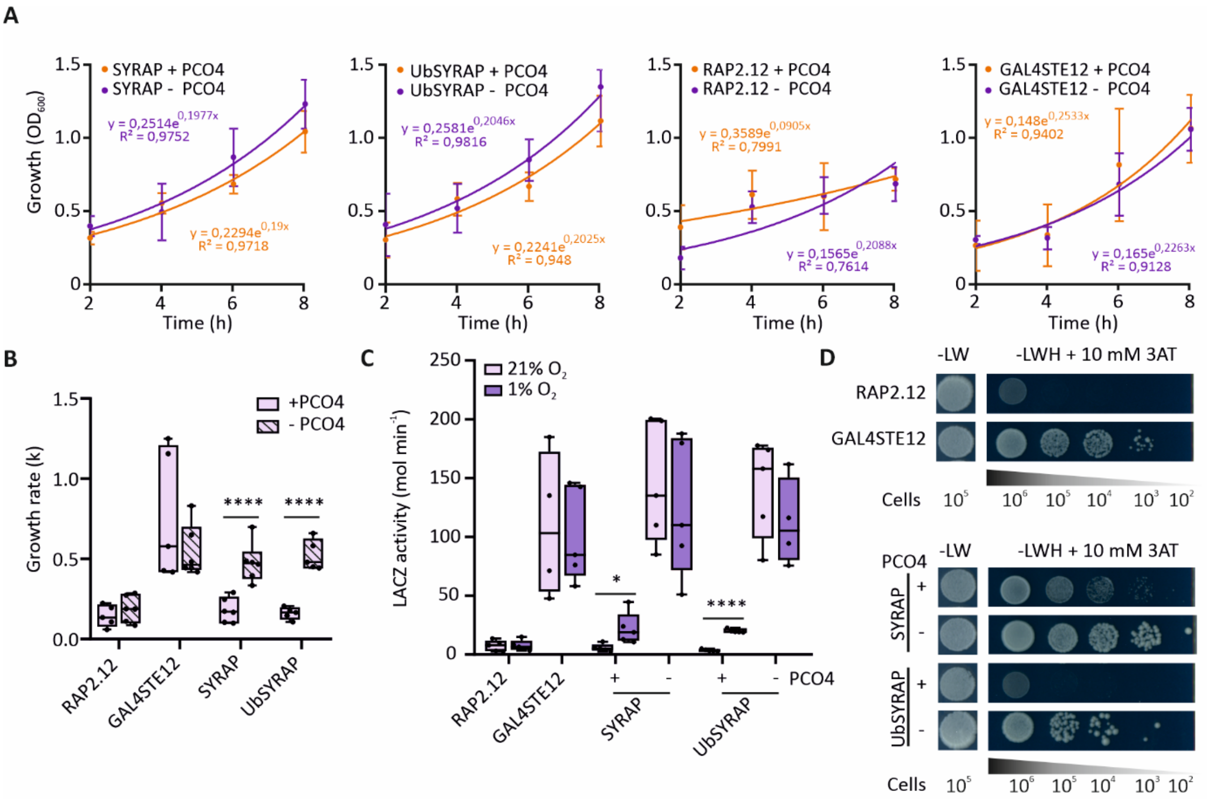

Figure S6. Orthogonal Cys-NDP regulation in MaV203 strain yeast. **(A)** Growth curves (OD_{600}) of aerated cultures expressing SYRAP or UbSYRAP, along with either PCO4 or a β-glucoronidase (GUS) protein (-PCO4). GAL4STE or RAP2.12 expressing colonies were also tested as positive or negative controls, respectively. GAL4STE can autonomously bind and activate UAS_{GAL}, while RAP2.12 lacks a GAL4-BD domain. Exponential regressions were fitted to the data, displayed as mean ± SD (n=5). **(B)** Growth rate constants (k) calculated between t_6 and t_4 from (A). **(C)** LACZ activity in liquid cultures after 6 h hypoxic treatment. LACZ activity was measured through colorimetric CPRG assay. **(D)** Aerobic growth of spotted colonies on –leu-his-trp (-LWH) solid media, supplemented with 10 mM 3-AT. Ten-fold serial dilutions were made, resulting in the approximate cell number indicated below. Asterisks represent statistically different groups, as tested via paired Student's t-tests, for +PCO4 vs. -PCO4 (B) and air vs. hypoxia (C); (n=4-6; ****, p<0.0001; *, p<0.05)

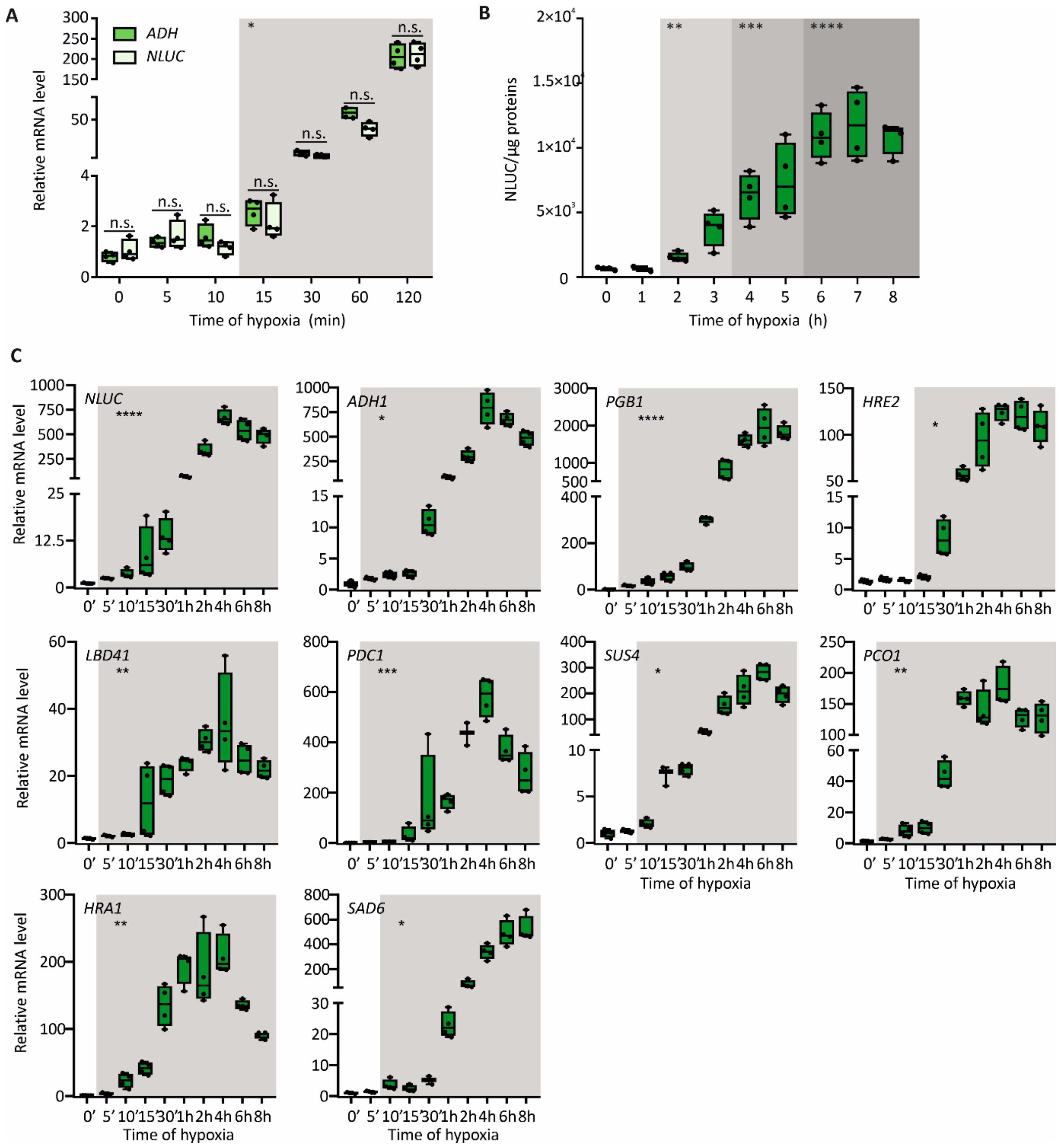

Figure S7. Characterization of *NLUC* expression and long-term activity in the HRPE reporter line of Arabidopsis. (A) Relative expression to a normoxic sample of *ADH1* and *NLUC,* normalized to *UBQ10*. Paired Student's t-tests for each time-point were performed (n=4; n.s., p≥0.05). **(B)** Long-term NLUC activity, normalized on total protein content, in seedlings exposed to a time course of hypoxia. **(C)** Extended profiling of HRG expression in the same plants as (B). All experiments were carried out with 7-day-old $HRPE_{ADH}$-*NLUC* seedlings grown on vertical plates. Asterisks and shading indicate statistically significant differences after multiple Student's t-tests between each time-point and t0 (n=3-4; ****, p<0.0001; ***, 0.0001≤p<0.001; **, 0.001≤p<0.01; *, p<0.05) ; in (C), only the first statistically different group was highlighted.

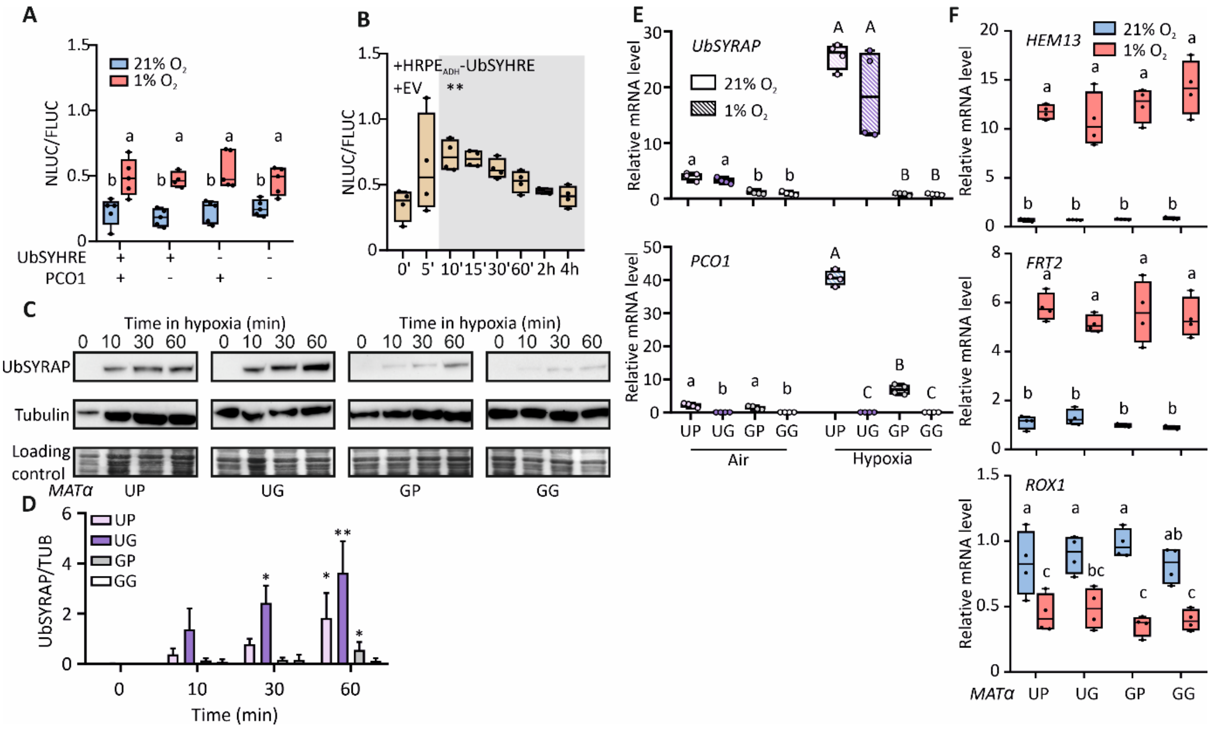

Figure S8. Characterization of the hypoxic feedback loop in yeast. (A) NLUC activity (normalized to P*GPD:FLUC*) in MynOx$^{MAT\alpha}$ cells mated with *MATα* cells containing combinations of $HRPE_{ADH}$-UbSYHRE, $HRPE_{ADH}$-PCO1 or $HRPE_{ADH}$-GUS ("-"). Cultures were incubated for 6 h in hypoxia or normoxia. **(B)** NLUC activity in diploid MynOx$^{MAT\alpha}$/$HRPE_{ADH}$-UbSYHRE$^{MAT\alpha}$ cells, growing on solid phase, challenged with up to 4 h hypoxia. **(C)** UbSYRAP protein abundance and **(D)** quantification (normalized to α-tubulin) (n=3 for 0, 10', 30' samples, n=4 for 60' samples) in the four diploid strains during a "hypoxic chase". Cultures were treated with 1% O_2 in pre-equilibrated media for up to 60 min. Replicate blots are provided in **SI Appendix, Figure S9.** Asterisks indicate significant differences after Student's t-test and pairwise comparisons between a given genotype and GG for the same time-point. Full statistical multiple analysis is provided in **Table S11**. **(E)** *UbSYRAP* and *PCO1* mRNA levels in diploid colonies before (21% O_2) or after 1 h hypoxia at 1% O_2. Different letters indicate statistically significant differences after One-way Anova in normoxic (lowercase) or hypoxic (uppercase) samples (n=4, $p<0.05$) with Benjamini, Krieger and Yekutieli correction. **(F)** Expression of yeast hypoxia marker genes in diploid colonies treated with 1% O_2 for 4 hours. In (A) and (F), letters indicate statistically significant differences after Two-way Anova (n=4-5, $p<0.05$) followed by Tukey post-hoc test.

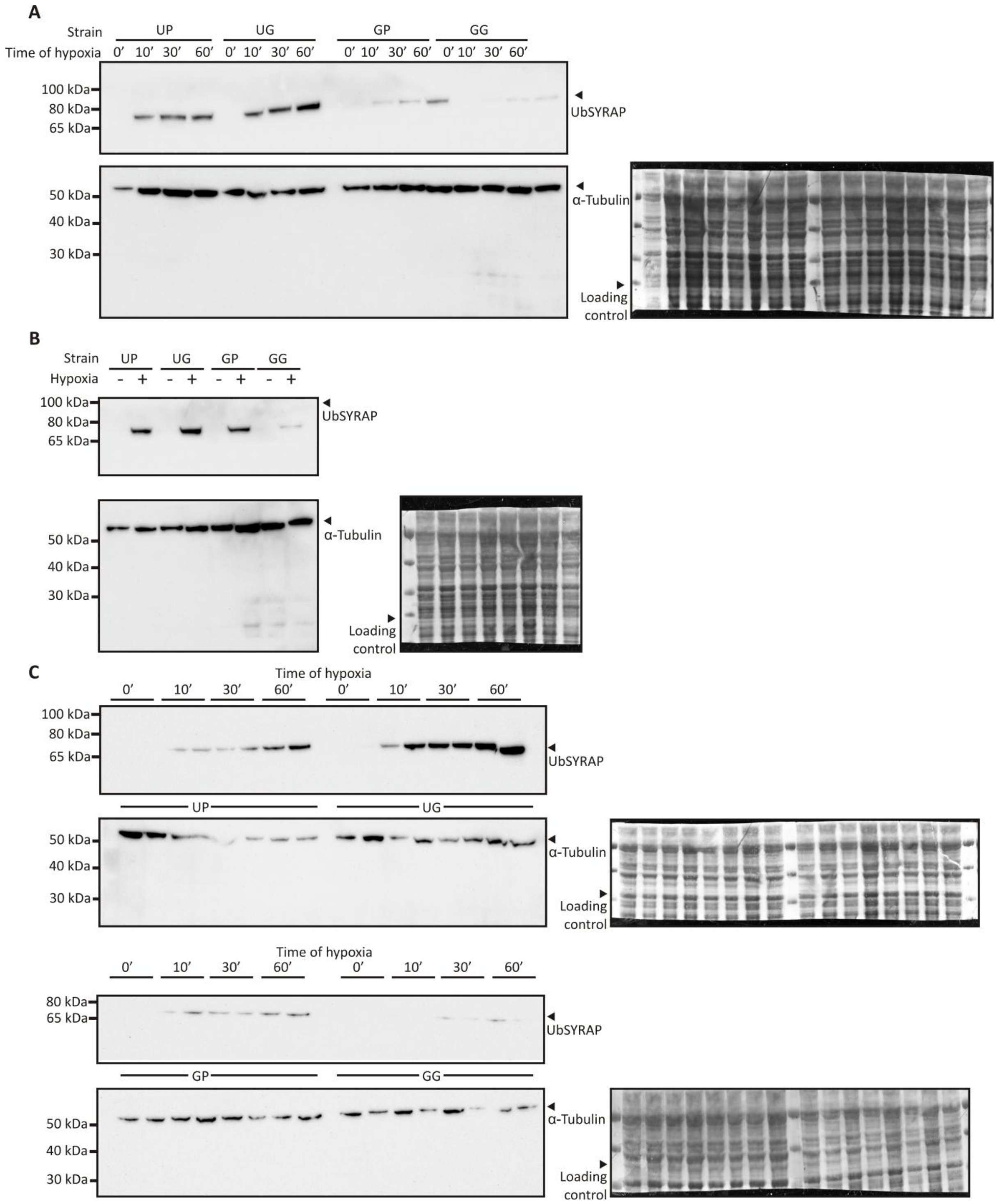

Figure S9. Supporting blots for Figure 5 and SI Appendix, Figure S8. UbSYRAP and α-tubulin protein abundance in MynOx$^{MAT\alpha}$ cells mated with *MATα* strains containing HRPE$_{ADH}$-UbSYRAP (U), HRPE$_{ADH}$-PCO1 (P) or HRPE$_{ADH}$-GUS (G) in UP, UG, GP and GG cells. Up to 3 blots **(A, B, C)** with different samples were used for quantification (n=3 for 10', 30' samples, additional blot in B, for 0 and 60' samples; n=4). Amido black stained membranes are shown besides the corresponding blots to reveal total protein loading.

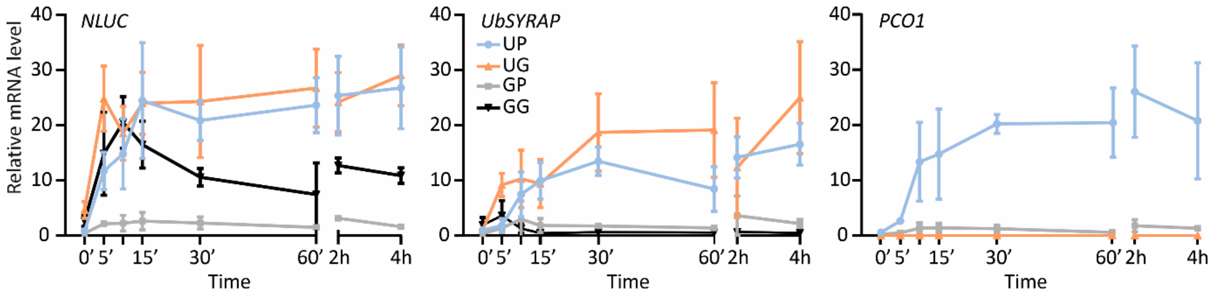

Figure S10. Solid-phase mRNA dynamics in yeast. Profiling of *NLUC, UbSYRAP* and *PCO1* expression in diploid cells grown in thin spots (pre-grown for 28 h on solid phase; n=4) over a 4 hour-long time course of hypoxia (1% O_2). Supporting statistics in **Table S12**.

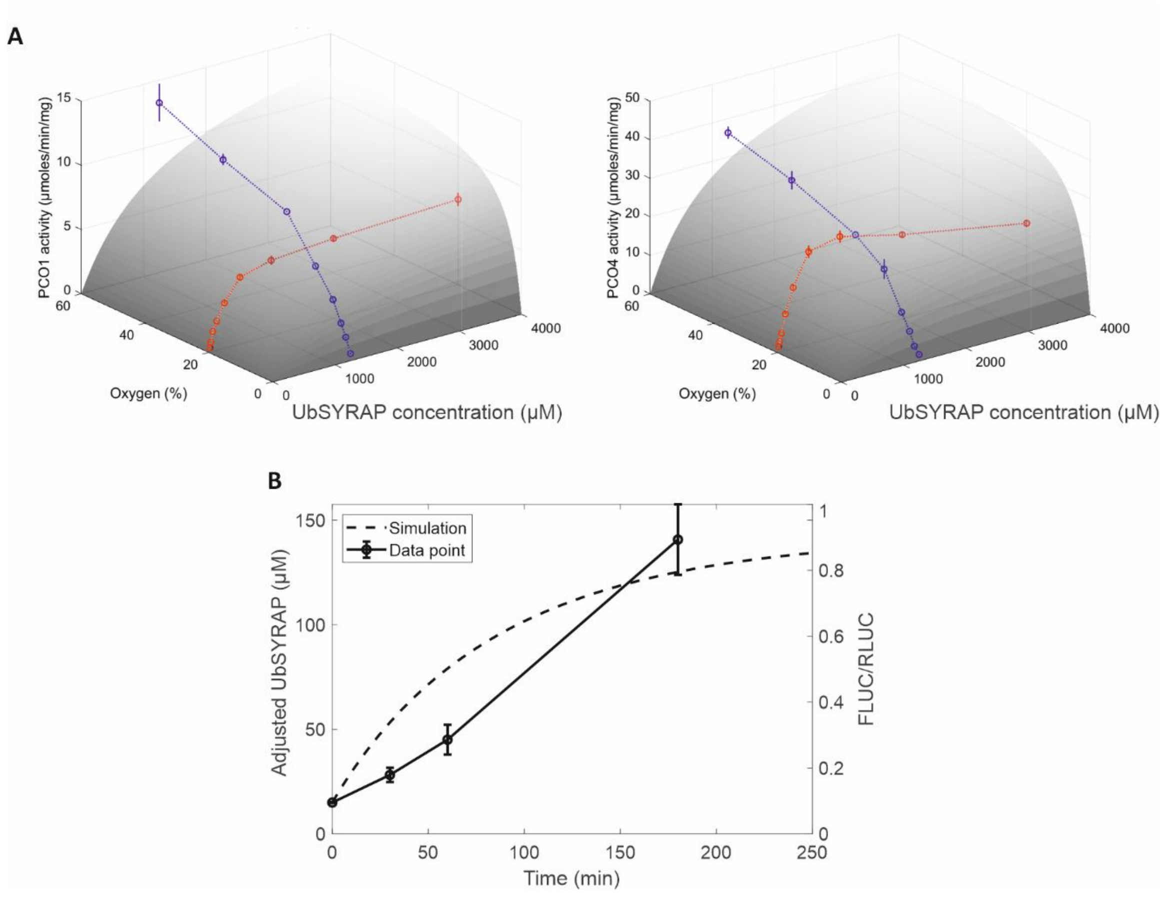

Figure S11. Kinetic parameters fitting and visualization. (A) 3-D data visualization and fitting for UbSYRAP and PCO1/4 kinetics. Kinetic data are from (13), considering RAP2.12 dynamics as a faithful approximation of UbSYRAP dynamics. Blue and red lines indicate PCO activity at different oxygen and UbSYRAP concentrations, respectively. **(B)** Kinetic parameters fitting for UbSYRAP synthesis rate by the *GPD* promoter and degradation rate in yeast. UbSYRAP concentration was rescaled based on published DLOR data from (12).

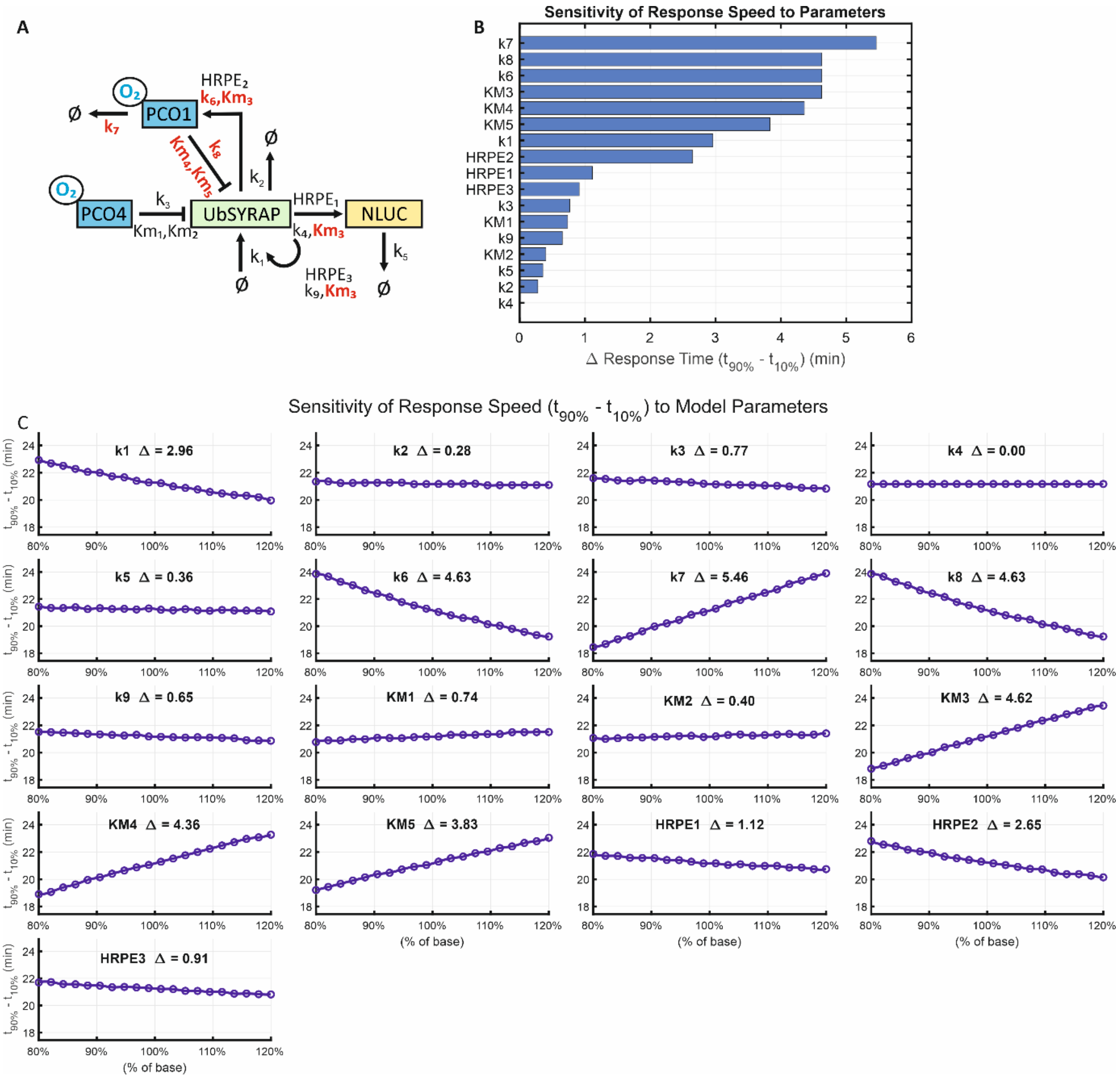

Figure S12. Response time sensitivity analysis to the model parameters. (A) UP model, highlighting in red the top 6 parameters affecting the model's Response time (RT; $t_{90\%}$-$t_{10\%}$). **(B)** Ranking of parameters affecting RT. **(C)** Single parameter sensitivity analysis. Parameters were iterated from 80% to 120% their fitted value (% of base).

Supporting Tables

Table S1. Parameters used for HRG expression equations. Parameters defining the logistic equation fitted into gene expression analysis of Fig. 1A (2 h hypoxia, 1% v/v O_2 in Col-0 seedlings growing in vertical plates).

Gene	Max. expression (A)	Rate constant (k) (min^{-1})	Half-max time (t_{half}) (min)
ADH1	326.32	0.064	74.87
PGB1	83.27	0.046	74.46
HRE2	219.14	0.076	51.46
SAD6	137.93	0.052	87.59
PCO1	127.09	0.092	31.94
HRA1	171.66	0.317	12.39
LBD41	229.40	0.302	11.26
PDC1	145.89	0.060	54.43
SUS4	103.73	0.068	63.71

Table S2. Residual analysis supporting Fig. 1B. Shown below, for each HRG studied in Fig. 1B, their fitted values, residual, studentized residual (St. Residual) leverage and Cooks' Distance (Cooks D.).

Gene	Fitted	Residual	St. Residual	Leverage	Cooks D.
PCO1	72.1	-24.5	-1.82	0.185	0.181
LBD41	29.4	-14.8	-1.03	0.330	0.174
PDC1	58.7	15.1	1.05	0.317	0.167
HUP7	57.3	26.5	1.93	0.112	0.107
HRA1	11.4	2.51	0.263	0.752	0.083
HB1	91.2	3.57	0.278	0.549	0.037
HRE2	66.2	-8.4	-0.522	0.263	0.037
SUS4	66.8	-2.49	-0.149	0.248	0.003
ADH1	66.1	2.49	0.149	0.244	0.003

Table S3. Ordinary differential equations of the UbSYRAP and PCO1/4 kinetic model. The reaction rates v1 - v9 are given in Table S3.

	Left-hand Sides	Right-hand Sides	Initial Concentrations (Simulated)
GG model	d[UbSYRAP]/dt	v1-v2-v3	9.1087 µM
	d[NLUC/FLUC]/dt	v4-v5	0.2402
GP model	d[UbSYRAP]/dt	v1-v2-v3-v8	3.9663 µM
	d[NLUC/FLUC]/dt	v4-v5	0.1800
	d[PCO1]/dt	v6-v7	0.2450 µM
UG model	d[UbSYRAP]/dt	v1-v2-v3+v9	10.2490 µM
	d[NLUC/FLUC]/dt	v4-v5	0.2744
UP model	d[UbSYRAP]/dt	v1-v2-v3-v8+v9	7.9160 µM
	d[NLUC/FLUC]/dt	v4-v5	0.2116
	d[PCO1]/dt	v6-v7	0.0082 µM

Table S4. Reaction rates of the UBSYRAP and PCO1/4 kinetic model. Null indicates degradation.

	Reactions	Reaction Rates	Description
v_1	→ UbSYRAP	k_1	UbSYRAP synthesis
v_2	UbSYRAP → null	$k_2 \cdot [\mathrm{UbSYRAP}]$	UbSYRAP degradation
v_3	UbSYRAP + O_2 + PCO4 → PCO4	$k_3 \cdot [\mathrm{PCO4}] \cdot \frac{[O_2]}{Km_1 + [O_2]} \cdot \frac{[\mathrm{UbSYRAP}]}{Km_2 + [\mathrm{UbSYRAP}]}$	UbSYRAP degradation via NDP by PCO4
v_4	UbSYRAP + $HRPE_1$ → NLUC/FLUC + UbSYRAP + $HRPE_1$	$k_4 \cdot \frac{[\mathrm{UbSYRAP}] \cdot HRPE_1}{Km_3 + [\mathrm{UbSYRAP}] \cdot HRPE_1}$	NLUC/FLUC production by UbSYRAP binding to HRPE
v_5	NLUC/FLUC → null	$k_5 \cdot [\mathrm{NLUC/FLUC}]$	NLUC/FLUC degradation
v_6	UbSYRAP + $HRPE_2$ → PCO1 + UbSYRAP + $HRPE_2$	$k_6 \cdot \frac{[\mathrm{UbSYRAP}] \cdot HRPE_2}{Km_3 + [\mathrm{UbSYRAP}] \cdot HRPE_2}$	PCO1 synthesis via feedback loop
v_7	PCO1 → null	$k_7 \cdot [\mathrm{PCO1}]$	PCO1 degradation
v_8	UbSYRAP + O_2 + PCO1 → PCO1	$k_8 \cdot [\mathrm{PCO1}] \cdot \frac{[O_2]}{Km_4 + [O_2]} \cdot \frac{[\mathrm{UbSYRAP}]}{Km_5 + [\mathrm{UbSYRAP}]}$	UbSYRAP degradation via NDP by PCO1
v_9	UbSYRAP + $HRPE_3$ → UbSYRAP + UbSYRAP + $HRPE_3$	$k_9 \cdot \frac{[\mathrm{UbSYRAP}] \cdot HRPE_3}{Km_3 + [\mathrm{UbSYRAP}] \cdot HRPE_3}$	UbSYRAP synthesis via feedback loop

Table S5. Parameter values used in the UbSYRAP and PCO1/4 kinetic model. Concentrations and the Michaelis-Menten constants (Kms) are given in µM, except Kms for O_2 are in %. Rate constants (ks) are expressed in s^{-1}, except k_1, k_6 and k_9 are in µM s^{-1}.

Parameters	Description	Values	References
k_1	UbSYRAP protein synthesis rate	0.0301	Fitted (Puerta et al., 2019)
k_2	UbSYRAP degradation rate	0.0001	Fitted (Puerta et al., 2019)
k_3	Catalytic rate constant for PCO4-mediated oxidation of UbSYRAP	26.5878	Re-fitted (White et al., 2018)
K_{m1}	Michaelis-Menten constant for O_2 as a substrate of PCO4	16.4217	Re-fitted (White et al., 2018)
K_{m2}	Michaelis-Menten constant for UbSYRAP as a substrate of PCO4	270.2505	Re-fitted (White et al., 2018)
$PCO4_{GG}$	PCO4 protein concentration	0.06	Fitted
$PCO4_{GP}$		0.06	Fitted
$PCO4_{UG}$		0.08	Fitted
$PCO4_{UP}$		0.08	Fitted
k_{4GG}	NLUC protein synthesis rate constant measured by UbSYRAP activating $HRPE_1$.	0.06	Fitted
k_{4GP}		0.0067	Fitted
k_{4UG}		0.0067	Fitted
k_{4UP}		0.0088	Fitted
$HRPE_{1GG}$	Binding rate of UbSYRAP to the promoter containing 5xHRPE for NLUC/FLUC in GG, GP, UG, and UP models	0.07	Fitted
$HRPE_{1GP}$		0.06	Fitted
$HRPE_{1UG}$		0.04	Fitted
$HRPE_{1UP}$		0.02	Fitted
$HRPE_{2GP}$	Binding rate of UbSYRAP to the promoter containing 5xHRPE for PCO1 synthesis in GP and UP models	0.01	Fitted
$HRPE_{2UP}$		0.005	Fitted
$HRPE_{3UG}$	Binding rate of UbSYRAP to the promoter containing 5xHRPE for UbSYRAP synthesis in UG and UP models	0.02	Fitted
$HRPE_{3UP}$		0.015	Fitted
Km_3	Michaelis-Menten constant for HRPE activation by UbSYRAP	0.5	Fitted
k_{5GG}	NLUC/FLUC decay rate	0.014	Fitted
k_{5GP}		0.012	Fitted
k_{5UG}		0.011	Fitted
k_{5UP}		0.010	Fitted
k_{6GP}	PCO1 protein synthesis rate constant measured by UbSYRAP activating $HRPE_2$.	0.0002	Fitted
k_{6UP}		0.003	Fitted
k_7	PCO1 degradation rate	0.0009	Fitted
k_8	Catalytic rate constant for PCO1-mediated oxidation of UbSYRAP	9.6688	Re-fitted (White et al., 2018)
K_{m4}	Michaelis-Menten constant for O_2 as a substrate of PCO1	19.5138	Re-fitted (White et al., 2018)
K_{m5}	Michaelis-Menten constant for UbSYRAP as a substrate of PCO1	286.6942	Re-fitted (White et al., 2018)
k_{9UG}	UbSYRAP protein synthesis rate constant measured by UbSYRAP activating $HRPE_3$.	0.05	Fitted
k_{9UP}		0.03	Fitted

Table S6. *S. cerevisiae* strains generated in this work. Name, genotype detailing the expression plasmid's integration site and selection media (Y: YPD; SD-4: SD -his-trp-ura-leu). In bold, wild-type strains. In MaV203, bold indicates the integrated UAS_{GAL} reporters.

Strain name	Genotype	Media
**MaV203**	*MATα; leu2-3,112; trp1-901; his3Δ200; ade2-101; cyh2^R; can1^R; gal4Δ; gal80Δ;* ***GAL1-lacZ; HIS3$_{UASGAL1}$-HIS3@LYS2; SPAL10$_{UASGAL1}$-URA3***	Y
**W3031A**	*MATα; his3-11_15; leu2-3_112; ura3-1; trp1Δ2; ade2-1; can1-100*	Y
**W3031B**	*MATα; his3-11_15; leu2-3_112; ura3-1; trp1Δ2; ade2-1; can1-100*	Y
MGUS	*MaV203 leu2::LEU2 pAG305GPD-GUS*	SD - leu
MPCO	*MaV203 leu2::LEU2 pAG305GPD-PCO4-SV40*	SD - leu
MGUS|R	*MGUS trp1::TRP1 pAG304GPD-RAP2.12*	SD - trp-leu
MGUS|G4S	*MGUS trp1::TRP1 pAG304GPD-GAL4Ste12*	SD - trp-leu
MGUS|SYRAP	*MGUS trp1::TRP1 pAG304GPD-SYRAP*	SD - trp-leu
MPCO|SYRAP	*MPCO trp1::TRP1 pAG304GPD-SYRAP*	SD - trp-leu
MGUS|UbSYRAP	*MGUS trp1::TRP1 pAG304GPD-UbSYRAP*	SD - trp-leu
MPCO|UbSYRAP	*MPCO trp1::TRP1 pAG304GPD- UbSYRAP*	SD - trp-leu
MDLOR|PCO.1	*MaV203 trp1::TRP1 pAG304GPD-C-DLOR + pAG415GPD-PCO4*	SD - trp-leu
MDLOR|PCO.2	*MaV203 trp1::TRP1 pAG304GPD-C-DLOR + pAG415GPD-PCO4-SV40*	SD - trp-leu
WFLUC	*W3031A his3::HIS3 pAG303GPD-FLUC*	SD - his
WH	*WFLUC trp1::TRP1 pAG304GPD-HRPE-NLUC*	SD - trp-his
WHO	*WFLUC trp1::TRP1 pAG304GPD-HRPE$_{Ω}$-NLUC*	SD - trp-his
WHA	*WFLUC trp1::TRP1 pAG304GPD-HRPE$_{ADH}$-NLUC*	SD - trp-his
WH|R	*WH ura3::pAG306-RAP2.12*	SD - trp-ura-his
WH|G4S	*WH ura3::pAG306-G4Ste*	SD - trp-ura-his
WH|RG4	*WH ura3::URA3 pAG306GPD-RAP2.12-G4AD*	SD - trp-ura-his
WH|R6T	*WH ura3::URA3 pAG306GPD-RAP2.12-6TVP*	SD - trp-leu-his
WH|SYRAP	*WH ura3::URA3 pAG306GPD-SYRAP*	SD - trp-ura-his
WH|UbSYRAP	*WH ura3::URA3 pAG306GPD-UbSYRAP*	SD - trp-ura-his
WHO|R	*WHO ura3::pAG306-RAP2.12*	SD - trp-ura-his
WHO|G4S	*WHO ura3::pAG306-G4Ste*	SD - trp-ura-his
WHO|RG4	*WHO ura3::URA3 pAG306GPD- RAP2.12-G4AD*	SD - trp-ura-his
WHO|R6T	*WHO ura3::URA3 pAG306GPD- RAP2.12-6TVP*	SD - trp-ura-his
WHO|SYRAP	*WHO ura3::URA3 pAG306GPD-SYRAP*	SD - trp-ura-his
WHO|UbSYRAP	*WHO ura3::URA3 pAG306GPD-UbSYRAP*	SD - trp-ura-his
WHA|R	*WHA ura3::pAG306-RAP2.12*	SD - trp-ura-his
WHA|G4S	*WHA ura3::pAG306-G4Ste*	SD - trp-ura-his
WHA|RG4	*WHA ura3::URA3 pAG306GPD- RAP2.12-G4AD*	SD - trp-ura-his
WHA|R6T	*WHA ura3::URA3 pAG306GPD- RAP2.12-6TVP*	SD - trp-ura-his
WHA|SYRAP	*WHA ura3::URA3 pAG306GPD-SYRAP*	SD - trp-ura-his
WHA|UbSYRAP	*WHA ura3::URA3 pAG306GPD-UbSYRAP*	SD - trp-ura-his
WH|SYRAP|PCO	*WH|SYRAP leu2::LEU2 pAG305GPD-PCO4-SV40*	SD-4
WH|SYRAP|GUS	*WH|SYRAP leu2::LEU2 pAG305GPD-GUS*	SD-4
WH|UbSYRAP|PCO	*WH|UbSYRAP leu2::LEU2 pAG305GPD-PCO4-SV40*	SD-4
WH|UbSYRAP|GUS	*WH|UbSYRAP leu2::LEU2 pAG305GPD-GUS*	SD-4
WHA|SYRAP|PCO	*WHA|SYRAP leu2::LEU2 pAG305GPD-PCO4-SV40*	SD-4
WHA|SYRAP|GUS	*WHA|SYRAP leu2::LEU2 pAG305GPD-GUS*	SD-4
WHA|UbSYRAP|GUS	*WHA|UbSYRAP leu2::LEU2 pAG305GPD-GUS*	SD-4
WHA|UbSYRAP|PCO **(MynOx)**	*WHA|UbSYRAP leu2::LEU2 pAG305GPD-PCO4-SV40*	SD-4
**W303 *MATα* strains**	**Genotype**	**Media**
WB|H_AUbSYRAP	*W3031B his3::HIS3 pAG303HRPE$_{ADH}$-UbSYRAP*	SD-4
WB|H_AGUS	*W3031B his3::HIS3 pAG303HRPE$_{ADH}$-GUS*	SD-4
WB|H_AUbSYHRE	*W3031B his3::HIS3 pAG303HRPE$_{ADH}$-UbSYHRE*	
WB|H_AUbSYRAP|H_APCO	*WB|H_AUbSYRAP leu2::LEU2 Pag305HRPE$_{ADH}$-PCO1-GFP*	SD-4

WB|H_AUbSYRAP|H_AGUS	*WB|H_AUbSYRAP leu2::LEU2 Pag305HRPE$_{ADH}$-GUS*	SD-4
WB|H_AGUS|H_APCO	*WB|H_AGUS leu2::LEU2 Pag305HRPE$_{ADH}$-PCO1-GFP*	SD-4
WB|H_AGUS|H_AGUS	*WB|H_AGUS leu2::LEU2 Pag305HRPE$_{ADH}$-GUS*	SD-4
WB| H_AUbSYHRE |H_APCO	*WB|H_AUbSYHRE leu2::LEU2 Pag305HRPE$_{ADH}$-PCO1*	SD-4
WB| H_AUbSYHRE |H_AGUS	*WB|H_AUbSYHRE leu2::LEU2 Pag305HRPE$_{ADH}$-GUS*	SD-4
**Mated strains**	**Genotype**	**Media**
MynOx x UP	*MynOx x WB|H_AUbSYRAP|H_APCO*	SD-4
MynOx x UG	*MynOx x WB|H_AUbSYRAP|H_AGUS*	SD-4
MynOx x GP	*MynOx x WB|H_AGUS|H_APCO*	SD-4
MynOx x GG	*MynOx x WB|H_AGUS|H_AGUS*	SD-4
MynOx x HRE2P	*MynOx x WB| H_AUbSYHRE |H_APCO*	SD-4
MynOx x HRE2G	*MynOx x WB| H_AUbSYHRE |H_AGUS*	SD-4

Table S7. Synthetic sequences used in this study. Color code for NLUC-UBQ-RAP2.12$_{2-50}$-GAL4STE string: NLUC (Orange), UBQ (Black), RAP2.12 N-terminal fragment corresponding to aa 2-50 (Green), Gal4Ste (Purple for Gal4- and Blue for Ste-derived sequences). For UbSYRAP: UBQ (black), full RAP2.12 coding sequence devoid of initial Met codon (green) and GAL4Ste (purple/blue), flanked by attB sites compatible for BP cloning reaction (red). For ccdB-NLUC: SacI restriction site (bold), attL1 (pink), ccdB cassette with chloramphenicol resistance gene (underlined) and ccdb toxin (light blue), attL2 (pink), NLUC (orange), XhoI restriction site (bold).

String name	Sequence (5'-3')
NLUC-UBQ-RAP2.12$_{2-50}$-GAL4STE	ATGGTTTTCACCCTTGAGGACTTCGTTGGAGATTGGAGACAGACCGCTGGATACAACCTTGATCAGG<br>TGTTGGAGCAAGGTGGGGTGTCATCTTTGTTCCAGAACCTCGGAGTTAGCGTGACCCCTATCCAGAG<br>AATCGTTCTCTCTGGTGAGAACGGGCTCAAGATCGATATCCACGTGATCATCCCTTACGAGGGACTT<br>AGCGGAGATCAGATGGGACAGATCGAGAAGATTTTCAAGGTGGTGTACCCTGTGGACGACCACCACT<br>TCAAGGTTATCCTCCATTACGGAACCCTCGTGATCGATGGTGTGACCCCAAACATGATCGACTACTT<br>CGGTAGACCGTACGAGGGAATCGCTGTGTTCGATGGAAAGAAGATTACCGTCACTGGGACCCTCTGG<br>AACGGGAACAAGATTATCGATGAGAGGCTCATCAACCCGGACGGCTCACTTTTGTTCAGAGTGACTA<br>TCAACGGTGTGACCGGTTGGAGACTTTGCGAGAGAATTTTGGCTATGCAAATTTTCGTGAAAACACT<br>CACTGGTAAGACCATCACT**CTCGAG**GTTGAGAGCTCTGACACCATCGACAATGTTAAGGCAAAGATC<br>CAGGACAAGGAAGGCATTCCTCCTGACCAGCAAAGATTGATCTTCGCTGGTAAGCAGCTAGAAGATG<br>GCCGCACCTTGGCTGATTACAACATCCAGAAAGAATCAACACTCCACTTGGTTCTCAGGTTAAGAGG<br>TGGTTGTGGGGGAGCTATCATTTCTGATTTCATCTGGTCGAAATCTGAGTCAGAACCGAGTCAACTC<br>GGCTCTGTTAGCAGCAGGAAGAAGCGTAAACCCGTCTCAGTGAGTGAAGAAAGAGATGGAAACGAG<br>AGAGGAAGAATCTGTACATGAAGCTGTTGTCCTCTATTGAACAAGCTTGCGATATCTGCAGGCTGAA<br>AAAATTGAAGTGCTCCAAAGAAAAGCCAAAGTGTGCTAAGTGTTTGAAGAACAACTGGGAATGTAGA<br>TACTCTCCAAAGACTAAGAGATCTCCATTGACTAGAGCACATTTGACCGAAGTTGAATCTAGGTTGG<br>AAAGATTGGAGCAGCTGTTCTTGTTGATTTTCCCAAGAGAAGATCTGGACATGATCTTGAAGATGGA<br>TTCCTTGCAAGATATCAAGGCTTTGTTGACTGGTTTGTTCGTTCAAGATAACGTTAACAAGGATGCC<br>GTTACTGATAGATTGGCTTCTGTTGAAACTGATATGCCATTGACCTTGAGACAACATAGAATTTCTG<br>CTACCTCCAGCTCTGAAGAATCTTCTAACAAAGGTCAAAGACAGTTGACCGTGTCTAGAGATGAAGA<br>AGATTTTCCATTGGATTACTTCCCAGTCTCTGTTGAATATCCAACTGAAGAAAATGCCTTCGATCCA<br>TTTCCACCACAAGCTTTTACTCCAGCTATTGATTCTGCTGCTCATCATGATAACTCTACTATCCCAT<br>TAGACTTCATGCCAAGAGATGCATTGCATGGTTTTGATTGGTCTGAAGAGGATGATATGTCTGATGG<br>TTTGCCATTCTTGAAAACCGATCCAAACAACAACGGTTTCTAA
UbSYRAP	GGGGACAAGTTTGTACAAAAAAGCAGGCTCCATGCAAATTTTCGTGAAAACACTCACTGGTAAGACC<br>ATCACT**CTCGAG**GTTGAGAGCTCTGACACCATCGACAATGTTAAGGCAAAGATCCAGGACAAGGAAG<br>GCATTCCTCCTGACCAGCAAAGATTGATCTTCGCTGGTAAGCAGCTAGAAGATGGCCGCACCTTGGC<br>TGATTACAACATCCAGAAAGAATCAACACTCCACTTGGTTCTCAGGTTAAGAGGTGGTTGTGGAGGA<br>GCTATAATATCCGATTTCATTCCACCGCCGAGGTCTCGCCGTGTTACTAGCGAGTTTATTTGGCCGG<br>ATCTGAAGAAGAATTTGAAAGGATCGAAGAAAAGCTCGAAGAATCGTTCGAATTTCTTCGATTTTGA<br>CGCTGAGTTCGAAGCTGATTTCCAAGGTTTCAAAGATGATTCGTCTATCGATTGCGATGATGATTTC<br>GACGTCGGTGATGTTTTCGCCGATGTGAAACCATTCGTTTTCACTTCGACTCCAAAACCCGCCGTCT<br>CCGCCGCTGCGGAAGGTTCAGTTTTTGGTAAGAAAGTTACTGGCTTGGATGGGGACGCTGAGAAATC<br>TGCAAATAGGAAGAGGAAGAATCAGTACCGAGGGATTAGGCAACGTCCTTGGGGAAAATGGGCTGCT<br>GAGATACGTGATCCAAGGGAAGGTGCTAGAATCTGGCTTGGAACGTTCAAGACAGCTGAGGAAGCTG<br>CTAGAGCTTACGATGCTGCAGCGCGGAGAATCCGTGGATCTAAAGCTAAGGTGAATTTCCCTGAAGA<br>AAACATGAAGGCTAATTCTCAGAAACGCTCTGTGAAGGCTAATCTTCAGAAACCAGTGGCTAAACCT<br>AACCCTAACCCAAGTCCAGCTTTGGTTCAGAACTCGAACATCTCCTTTGAAAATATGTGTTTCATGG<br>AGGAGAAACACCAAGTGAGCAACAACAACAACAACCAGTTTGGGATGACAAACTCCGTTGATGCTGG<br>ATGTAATGGGTATCAGTATTTCAGCTCTGACCAGGGTAGTAATTCTTTCGATTGTTCGGAGTTTGGT<br>TGGAGCGATCAAGCTCCGATAACTCCCGACATCTCTTCTGCGGTTATCAACAACAACAACTCAGCTC<br>TGTTCTTTGAGGAAGCCAATCCAGCTAAGAAGCTCAAGTCTATGGATTTCGAGACACCTTACAACAA<br>CACTGAATGGGACGCTTCACTGGATTTCCTCAACGAAGATGCTGTAACGACTCAGGACAATGGTGCA<br>AACCCTATGGACCTATGGAGTATTGATGAAATTCATTCCATGATTGGAGGAGTCTTCATGAAGCTGT<br>TGTCCTCTATTGAACAAGCTTGCGATATCTGCAGGCTGAAAAAATTGAAGTGCTCCAAAGAAAAGCC<br>AAAGTGTGCTAAGTGTTTGAAGAACAACTGGGAATGTAGATACTCTCCAAAGACTAAGAGATCTCCA<br>TTGACTAGAGCACATTTGACCGAAGTTGAATCTAGGTTGGAAAGATTGGAGCAGCTGTTCTTGTTGA<br>TTTTCCCAAGAGAAGATCTGGACATGATCTTGAAGATGGATTCCTTGCAAGATATCAAGGCTTTGTT<br>GACTGGTTTGTTCGTTCAAGATAACGTTAACAAGGATGCCGTTACTGATAGATTGGCTTCTGTTGAA<br>ACTGATATGCCATTGACCTTGAGACAACATAGAATTTCTGCTACCTCCAGCTCTGAAGAATCTTCTA<br>ACAAAGGTCAAAGACAGTTGACCGTGTCTAGAGATGAAGAAGATTTTCCATTGGATTACTTCCCAGT<br>CTCTGTTGAATATCCAACTGAAGAAAATGCCTTCGATCCATTTCCACCACAAGCTTTTACTCCAGCT<br>ATTGATTCTGCTGCTCATCATGATAACTCTACTATCCCATTAGACTTCATGCCAAGAGATGCATTGC<br>ATGGTTTTGATTGGTCTGAAGAGGATGATATGTCTGATGGTTTGCCATTCTTGAAAACCGATCCAAA<br>CAACAACGGTTTCTAAcacccagctttcTTGTACAAAGTGGTCCCC

ccdB-NLUC	**gagctcACAAGTTTGTACAAAAAAGCTGAACGAGAAACGTAAAATGATATAAATATCAATATATTAA**<br>**ATTAGATTTTGCATAAAAAACAGACTACATAATACTGTAAAACACAACATATCCAGTCACTATG**GCG<br>GCCGCATTAGGCACCCCAGGCTTTACACTTTATGCTTCCGGCTCGTATAATGTGTGGATTTTGAGTT<br>AGGATCCGGCGAGATTTTCAGGAGCTAAGGAAGCTAAAATGGAGAAAAAAATCACTGGATATACCAC<br>CGTTGATATATCCCAATGGCATCGTAAAGAACATTTTGAGGCATTTCAGTCAGTTGCTCAATGTACC<br>TATAACCAGACCGTTCAGCTGGATATTACGGCCTTTTTAAAGACCGTAAAGAAAAATAAGCACAAGT<br>TTTATCCGGCCTTTATTCACATTCTTGCCCGCCTGATGAATGCTCATCCGGAATTCCGTATGGCAAT<br>GAAAGACGGTGAGCTGGTGATATGGGATAGTGTTCACCCTTGTTACACCGTTTTCCATGAGCAAACT<br>GAAACGTTTTCATCGCTCTGGAGTGAATACCACGACGATTTCCGGCAGTTTCTACACATATATTCGC<br>AAGATGTGGCGTGTTACGGTGAAAACCTGGCCTATTTCCCTAAAGGGTTTATTGAGAATATGTTTTT<br>CGTCTCAGCCAATCCCTGGGTGAGTTTCACCAGTTTTGATTTAAACGTGGCCAATATGGACAACTTC<br>TTCGCCCCCGTTTTCACCATGGGCAAATATTATACGCAAGGCGACAAGGTGCTGATGCCGCTGGCGA<br>TTCAGGTTCATCATGCCGTCTGTGATGGCTTCCATGTCGGCAGAATGCTTAATGAATTACAACAGTA<br>CTGCGATGAGTGGCAGGGCGGGGCGTAAACGCGTGGATCCGGCTTACTAAAAGCCAGATAACAGTAT<br>GCGTATTTGCGCGCTGATTTTTGCGGTATAAGAATATATACTGATATGTATACCCGAAGTATGTCAA<br>AAAGAGGTGTGCTATGAAGCAGCGTATTACAGTGACAGTTGACAGCGACAGCTATCAGTTGCTCAAG<br>GCATATATGATGTCAATATCTCCGGTCTGGTAAGCACAACCATGCAGAATGAAGCCCGTCGTCTGCG<br>TGCCGAACGCTGGAAAGCGGAAAATCAGGAAGGGATGGCTGAGGTCGCCCGGTTTATTGAAATGAAC<br>GGCTCTTTTGCTGACGAGAACAGGGACTGGTGAAATGCAGTTTAAGGTTTACACCTATAAAAGAGAG<br>AGCCGTTATCGTCTGTTTGTGGATGTACAGAGTGATATTATTGACACGCCCGGGCGACGGATGGTGA<br>TCCCCCTGGCCAGTGCACGTCTGCTGTCAGATAAAGTCTCCCGTGAACTTTACCCGGTGGTGCATAT<br>CGGGGATGAAAGCTGGCGCATGATGACCACCGATATGGCCAGTGTGCCGGTCTCCGTTATCGGGGAA<br>GAAGTGGCTGATCTCAGCCACCGCGAAAATGACATCAAAAACGCCATTAACCTGATGTTCTGGGGAA<br>TATAAATGTCAGGCTCCCTTATACACAGCCAGTCTGCAGGTCGAC**CATAGTGACTGGATATGTTGTG**<br>**TTTTACAGTATTATGTAGTCTGTTTTTTATGCAAAATCTAATTTAATATATTGATATTTATATCATT**<br>**TTACGTTTCTCGTTCAGCTTTCTTGTACAAAGTGG**TGATAAAAAA**ATGGTTTTCACCCTTGAGGACT**<br>**TCGTTGGAGATTGGAGACAGACCGCTGGATACAACCTTGATCAGGTGTTGGAGCAAGGTGGGGTGTC**<br>**ATCTTTGTTCCAGAACCTCGGAGTTAGCGTGACCCCTATCCAGAGAATCGTTCTCTCTGGTGAGAAC**<br>**GGGCTCAAGATCGATATCCACGTGATCATCCCTTACGAGGGACTTAGCGGAGATCAGATGGGACAGA**<br>**TCGAGAAGATTTTCAAGGTGGTGTACCCTGTGGACGACCACCACTTCAAGGTTATCCTCCATTACGG**<br>**AACCCTCGTGATCGATGGTGTGACCCCAAACATGATCGACTACTTCGGTAGACCGTACGAGGGAATC**<br>**GCTGTGTTCGATGGAAAGAAGATTACCGTCACTGGGACCCTCTGGAACGGGAACAAGATTATCGATG**<br>**AGAGGCTCATCAACCCGGACGGCTCACTTTTGTTCAGAGTGACTATCAACGGTGTGACCGGTTGGAG**<br>**ACTTTGCGAGAGAATTTTGGCTTAActcgag**

Table S8. Cloning primers used in this work. In bold, adaptors for BP cloning.

Function	Primer name	Sequence (5'-3')
PCO4-SV40 cloning	PCO4_BP_Fw	**AAAAAAGCAGGCTCC**ATGCCTTACTTTGCT
	PCO4_SV40_Rv1	CACTTTGCGTTTTTTTTTCGGAGTTCTAATGACA
	PCO4SV40_BP_Rv2	**AGAAAGCTGGGTG**TTACACTTTGCGTTTTT
RAP2.12	R2.12_Fw	**AAAAAAGCAGGCTCC**ATGTGTGGAGGAGCTA
RAP2.12-G4AD overlapping	OL_RAP_G4AD_Fw	ATTGGAGGAGTCTTCGCCAATTTTAATCAA
	OL_RAP_G4AD_Rv	TTGATTAAAATTGGCGAAGACTCCTCCAAT
	G4AD_BP_Rv	**AGAAAGCTGGGTG**TCACTCTTTTTTTGGGTTTG
RAP2.12-6TVP overlapping	OL_RAP_6TVP_Fw	GATTGGAGGAGTCTTCGGTGGTGGATCTGGTG
	OL_RAP_6TVP_Rv	CACCAGATCCACCACCGAAGACTCCTCCAATC
	6TVPSV40_BP_Rv	**AGAAAGCTGGGTG**TCATTACACTTTGCGTTTTTTTTTCGG
SYRAP overlapping	OL_R2.12-G4Ste_Fw	ATTGGAGGAGTCTTCATGAAGCTGTTGTCC
	OL_G4Ste – R2.12_Rv	GGACAACAGCTTCATGAAGACTCCTCCAAT
	G4Ste_Rv	**AGAAAGCTGGGTG**TTAGAAACCGTTGTTGTTT
UbSYRAP overlapping	SY_UBQ_Fw	**AAAAAAGCAGGCTCC**ATGCAAATTTTCGTGAAA
	OL_SY_Fw	AGGTTAAGAGGTGGTTGTGGAGGAGCTATA
	OL_SY_Rv	TATAGCTCCTCCACAACCACCTCTTAACCT
PCO1-GFP overlapping	PCO1gwFw	**AAAAAAGCAGGCTCC**ATGGGGTTTGAGATGAAACC
	PCO1GFP_OL_Fw	CCAAAGGTTGAAGATatggtgagcaagggc
	PCO1GFP_OL_Rv	gcccttgctcaccatATCTTCAACCTTTGG
	PCO1gwRv	**AGAAAGCTGGGTG**tcacttgtacagctcgtccatgc
UbSYHRE overlapping	UbSYHRE_UBgwFw	**AAAAAAGCAGGCTCC**ATGCCAACTTTGTACAAAAA
	UbSYHRE_HRG4OL_Fw	CAAGACTCCAATTAAATGAAGCTGTTGTCC
	UbSYHRE_HRG4OL_Rv	GGACAACAGCTTCATTTAATTGGAGTCTTG
	UbSYHRE_G4SgwRv	**AGAAAGCTGGGTG**TTAGAAACCGTTGTTGTTTGGAT
pAG303- and pAG305-HRPE$_{ADH}$ cloning	HRPE5xA30x_HF	GGCGAATTGGAGCTCTTCGAGTTTACCGCGGCCGCCCCCT
	HRPE5xA30x _p30xR	ATTCAGGTGAAGGGGGCGGCCGCGGTAAACTCGAAGAGCT
	HRPE30x_30xRv	CGTCGATCAATGTTGGATATCTAAACTCGAAGA
	HRPE5xA30x_p30xF	AAAAGCAAGTTCTTCACTGTTGATACGGATTCTAGAACTA
AttB1 adaptors	BP_Ad_Fw	GGGGACAAGTTTGTACAAAAAAGCAGGCT
	BP_Ad_Rv	GGGGACCACTTTGTACAAGAAAGCTGGGT

Table S9. Gateway-compatible vectors from this work. Inserts were recombined into the appropriate destination vectors from the list, as indicated in the main text, to generate the desired expression vectors. Descriptions of the destination vectors include auxotrophic markers, in the case of yeast expression plasmids, or selective markers for Agrobacteria/plants in the case of plant expression plasmids, and the features of the transgene expression cassette.

Entry vector	Insert	Source
HRPE_pE	5xHRPE	(15)
HRPE$_{\Omega}$_pE	5xHRPE-35SΩ leader	(15)
HRPE$_{ADH}$_pE	5xHRPE-ADH 5' UTR	(15)
GUS_pE	β-glucoronidase (GUS)	Thermo-Fisher Sci.
RAP2.12_pE	RAP2.12	(16)
PCO4_pE	PCO4	(12)
PCO1_pE	PCO1	(12)
C-DLOR_pE	RLUC-Ub-RAP2.12$_{2\text{-}28}$-FLUC	(12)
PCO4-SV40_201 (Addgene #249815)	PCO4 fused to the SV40 NLS	This study
G4Ste_201	GAL4$_{1\text{-}147}$-STE12$_{301\text{-}335}$Y310F-GAL4$_{148\text{-}196}$	This study
IB+_201	NLUC-AtUBQ4-RAP2.12$_{1\text{-}50}$-G4Ste	This study
SYRAP_201 (Addgene #249814)	RAP2.12-GAL4$_{1\text{-}147}$-G4Ste	This study
RAP2.12-G4AD_201	RAP2.12-GAL4AD	This study
RAP2.12-6TVP_201	RAP2.12-6XTAL-4XVP16	This study
UbSYRAP_201 (Addgene #249816)	AtUBQ4-RAP2.12$_{2\text{-}358}$-G4Ste	This study
UbSYHRE_201 (Addgene #249817)	AtUBQ4-HRE2$_{2\text{-}171}$-G4Ste	This study
**Destination vector**	**Description**	**Source**
pAG304GPD	Integrative plasmid TRP1, pGPD-ccdb-TCYC1	Addgene #14135
pAG305GPD	Integrative plasmid LEU2, pGPD-ccdb-TCYC1	Addgene #14138
pAG306GPD	Integrative plasmid URA3, pGPD-ccdb-TCYC1	Addgene #14140
pAG303GPD	Integrative plasmid HIS3, pGPD-ccdb-TCYC1	Addgene #14134
pAG304NLUC (Addgene #249736)	Integrative plasmid TRP1, ccdb-NLUC-TCYC1	This study
pK7WG2	Plant binary vector (Strep-Spec/Kan), p35S-ccdb-t35S	(4)
pBGWL7	Plant binary vector (Strep-Spec/Kan), ccdb-t35S	(4)
pAG303_HRPE$_{ADH}$ (Addgene #249735)	Integrative plasmid HIS3, HRPEADH-ccdb-TCYC1	This study
pAG305_HRPE$_{ADH}$ (Addgene #249736)	Integrative plasmid LEU2, HRPEADH-ccdb-TCYC1	This study
pBGWNLUCL7	Plant binary vector (Strep-Spec/Kan), ccdb-NLUC-t35S	This study

Table S10. qPCR primers used in this work. For the wild-type genes analyzed, the Arabidopsis Genome Initiative (AGI) identifier, or the yeast systematic code are given as codes.

Gene and code	Primer name	Sequence (5'-3')
AtUBQ10 (AT4G05320)	AtUBQ10_F	GGCCTTGTATAATCCCTGATGAATAAG
	AtUBQ10_R	AAAGAGATAACAGGAACGGAAACATAGT
AtADH1 (AT1G77120)	AtADH1_F	TATTCGATGCAAAGCTGCTGTG
	AtADH1_R	CGAACTTCGTGTTTCTGCGGT
AtPDC1 (AT4G33070)	AtPDC1_F	CACAGAATCTTCAATGCTTCTTACC
	AtPDC1_R	CCATGATAAAGCGTACATGGAA
AtPGB1 (AT2G16060)	AtPGB1_F	TTTGAGGTGGCCAAGTATGCA
	AtPGB1_R	TGATCATAAGCCTGACCCCAA
AtHRE2 (AT2G47520)	AtHRE2_F	GAAGCGTAAACCCGTCTCAGTG
	AtHRE2_R	TTTGCTCGGGYCACGAATCT
AtLBD41 (AT3G02550)	AtLBD41_F	TGAAGCGCAAGCTAACGCA
	AtLBD41_R	ATCCCAGGACGAAGGTGATTG
AtPCO1 (AT5G15120)	AtPCO1_F	ATTGGGTGGTTGATGCTCCAATG
	AtPCO1_R	ATGCATGTTCCCGCCATCTTC
AtSAD6 (AT1G43800)	AtSAD6_F	TTGTGGAAGGTGAAGGCAAGGG
	AtSAD6_R	TTGGCAACCCGCTTCTTTCTTACC
AtSUS4 (AT3G43190)	AtSUS4_F	CGCAGAACGTGTAATAACGCG
	AtSUS4_R	CAACCCTTGAGAGCAAAGCAAA
AtHRA1 (AT3G10040)	AtHRA1_F	ACAACCACCGCAACAGAATCC
	AtHRA1_R	TCTCCGCAATTCTCGCCAT
NLUC	NLUC_F	CCAGAACCTCGGAGTTAGCGTG
	NLUC_R	TCTGTCCCATCTGATCTCCGCT
ScACT1 (YFL039C)	ScACT1_F	CCATCCAAGCCGTTTTGTCC
	ScACT1_R	GGCGTGAGGTAGAGAGAAACC
UbSYRAP	UbSYRAP_F	AATGGGACGCTTCACTGGATTTC
	UbSYRAP_R	ATCGCAAGCTTGTTCAATAGAG
ScROX1 (YPR065W)	ScROX1_F	GTCCACAACTACCCCTACGC
	ScROX1_R	TAGCGGTGACCTCAGTGTTG
ScHEM13 (YDR044W)	ScHEM13_F	CACCAACTGCACAAGGATGC
	ScHEM13_R	CGTGTTTCCTTACGGTGGGT
ScCOX5B (YIL111W)	ScCOX5B_F	GATCTGCCCGAAAGATGGGAA
	ScCOX5B_R	CCACTCGCCGTAGGATATGT
ScFRT2 (YAL028W)	ScFRT2_F	TCGCATTCATCAGACGGGAG
	ScFRT2_R	GGCAGGAGAGGATTCGGAAG

Table S11. Statistical analysis supporting Fig. S8D. Student's t tests were performed on UbSYRAP/TUB band intensity and discovery determined by the Two-stage linear step-up procedure of Benjamini, Krieger and Yekutieli. P values of the analysis are displayed (in bold, p values < 0.05; n=3 for 0, 10' and 30', n=4 for 60').

	UP-UG	UP-GP	UP-GG	UG-GP	UG-GG	GP-GG
0'	0.528	0.557	0.336	0.717	0.969	0.526
10'	0.117	0.162	0.105	**0.062**	0.055	0.484
30'	**0.017**	**0.011**	0.051	**0.005**	**0.022**	0.911
60'	**0.066**	**0.052**	**0.015**	**0.003**	**0.001**	**0.037**

Table S12. Statistical analysis supporting Fig. S10. For each gene, Student's t tests were performed and discovery determined by the Two-stage linear step-up procedure of Benjamini, Krieger and Yekutieli. P values of the analysis are displayed (in bold, p<0.05, for n=4).

NLUC	UP-UG	UP-GP	UP-GG	UG-GP	UG-GG	GP-GG
0'	**0.003**	0.093	**0.013**	**0.002**	0.075	**0.004**
5'	**0.009**	**0.001**	0.483	**0.000**	0.105	**0.018**
10'	0.388	**0.009**	0.199	**0.001**	0.579	**0.000**
15'	0.938	**0.008**	0.217	**0.000**	0.076	**0.001**
30'	0.548	**0.000**	**0.002**	**0.005**	**0.037**	**0.000**
60'	0.498	**0.000**	**0.005**	**0.000**	**0.005**	0.083
2h	0.796	**0.003**	**0.013**	**0.001**	**0.006**	**0.000**
4h	0.639	**0.001**	**0.006**	**0.000**	**0.001**	**0.000**
PCO1	UP-UG	UP-GP	UP-GG	UG-GP	UG-GG	GP-GG
0'	**0.039**	0.198		0.055		
5'	**0.000**	**0.000**		**0.000**		
10'	**0.021**	**0.029**		**0.034**		
15'	**0.018**	**0.024**		**0.023**		
30'	**0.000**	**0.000**		**0.010**		
60'	**0.001**	**0.001**		**0.001**		
2h	**0.001**	**0.002**		**0.019**		
4h	**0.035**	**0.041**		**0.003**		
UbSYRAP	UP-UG	UP-GP	UP-GG	UG-GP	UG-GG	GP-GG
0'	0.958	0.062	0.253	**0.016**	0.237	0.070
5'	**0.003**	0.526	0.348	**0.000**	**0.018**	0.157
10'	0.429	0.137	0.253	**0.055**	**0.014**	0.359
15'	0.875	**0.004**	0.348	**0.019**	**0.008**	0.083
30'	0.216	**0.001**	0.024	**0.004**	**0.003**	**0.000**
60'	0.076	**0.013**	0.001	**0.007**	**0.006**	**0.051**
2h	0.718	**0.007**	0.001	0.120	**0.040**	0.152
4h	0.169	**0.001**	0.008	**0.004**	**0.002**	**0.003**

SI References

1. D. V Budescu, “Dominance Analysis: A New Approach to the Problem of Relative Importance of Predictors in Multiple Regression” (1993).
2. R. Azen, D. V. Budescu, The Dominance Analysis Approach for Comparing Predictors in Multiple Regression. *Psychol Methods* 8, 129–148 (2003).
3. R. D. Gietz, R. H. Schiestl, High-efficiency yeast transformation using the LiAc/SS carrier DNA/PEG method. *Nat Protoc* 2, 31–34 (2007).
4. M. Karimi, D. Inzé, A. Depicker, GATEWAY vectors for Agrobacterium-mediated plant.pdf. *Trends Plant Sci* 7, 193–195 (2002).
5. Z. Li, *et al.*, A potent Cas9-derived gene activator for plant and mammalian cells. *Nat Plants* 3, 930–936 (2017).
6. H. Pi, C. T. Chien, S. Fields, Transcriptional activation upon pheromone stimulation mediated by a small domain of Saccharomyces cerevisiae Ste12p. *Mol Cell Biol* 17, 6410–6418 (1997).
7. M. Lavilla-Puerta, *et al.*, Identification of novel plant cysteine oxidase inhibitors from a yeast chemical genetic screen. *Journal of Biological Chemistry* 299 (2023).
8. U. E. Akpan, P. G. Hugbo, R. N. Nasipuri, Kinetics of bacterial growth in simple intravenous solutions and admixtures. 14, 393–401 (1989).
9. D. A. Nielsen, J. Chou, A. J. MacKrell, M. J. Casadaban, D. F. Steiner, Expression of a preproinsulin-B-galactosidase gene fusion in mammalian cells. *Proceedings of the National Academy of Sciences* 80, 5198–5202 (1986).
10. T. von der Haar, Optimized protein extraction for quantitative proteomics of yeasts. *PLoS One* 2 (2007).
11. D. A. Weits, *et al.*, Plant cysteine oxidases control the oxygen-dependent branch of the N-end-rule pathway. *Nat Commun* 5, 1–10 (2014).
12. M. L. Puerta, *et al.*, A Ratiometric Sensor Based on Plant N-Terminal Degrons Able to Report Oxygen Dynamics in Saccharomyces cerevisiae. *J Mol Biol* 431, 2810–2820 (2019).
13. M. D. White, J. J. A. G. Kamps, S. East, L. J. Taylor Kearney, E. Flashman, The plant cysteine oxidases from Arabidopsis thaliana are kinetically tailored to act as oxygen sensors. *Journal of Biological Chemistry* 293, 11786–11795 (2018).
14. S. Alberti, A. D. Gitler, S. Lindquist, A suite of Gateway® cloning vectors for high-throughput genetic analysis in Saccharomyces cerevisiae. *Yeast* 24, 913–919 (2007).
15. G. Panicucci, S. Iacopino, E. De Meo, P. Perata, D. A. Weits, An Improved HRPE-Based Transcriptional Output Reporter to Detect Hypoxia and Anoxia in Plant Tissue. *Biosensors (Basel)* 10 (2020).
16. F. Licausi, *et al.*, Oxygen sensing in plants is mediated by an N-end rule pathway for protein destabilization. *Nature* 479, 419–422 (2011).
